# The TREM2 R47H variant is associated with liver-plasma-brain axis dyshomeostasis in the 5xFAD mouse model of Alzheimer’s disease

**DOI:** 10.1016/j.neurobiolaging.2026.04.007

**Published:** 2026-04-29

**Authors:** Gina Faraci, Benjamin Goodfriend, Joseph Bishop, Michael Vu, Julio Avelar-Barragan, Sage J.B. Dunham, Jason A. Rothman, Katrine L. Whiteson, Amrita K. Cheema, Giedre Milinkeviciute, Andrea J. Tenner, Frank M. LaFerla, Grant R. MacGregor, Kim N. Green, Mark Mapstone

**Affiliations:** a Department of Neurology, University of California, Irvine, CA 92617, USA; b Department of Molecular Biology and Biochemistry, University of California, Irvine, CA 92697, USA; c Institute for Memory Impairments and Neurological Disorders, University of California, Irvine, CA 92697, USA; d Department of Microbiology and Plant Pathology, University of California, Riverside, CA 92521, USA; e Department of Oncology, Georgetown University, Washington, DC 20007, USA; f Department of Neurobiology and Behavior, University of California, Irvine, CA 92697, USA; g Department of Pathology and Laboratory Medicine, University of California, Irvine, CA 92617, USA; h Transgenic Mouse Facility, ULAR, Office of Research, University of California, Irvine, CA 92697, USA; i Department of Developmental and Cell Biology, University of California, Irvine, CA 92697, USA

**Keywords:** Alzheimer’s disease, Metabolomics, Lipid, Mouse, Brain, Plasma, Liver

## Abstract

The human Triggering Receptor Expressed on Myeloid cells 2 (TREM2) gene is expressed predominantly by microglia in the brain and the R47H coding variant of *TREM2* is associated with increased risk for late-onset Alzheimer’s disease (LOAD). We performed lipidomic and metabolomic analysis of liver, plasma, and brain in 4- and 12-month-old *Trem2*^R47H^ homozygous (n = 27), 5xFAD hemizygous (n = 63), 5xFAD hemizygous, *Trem2*^R47H^ homozygous (n = 25), and wild type (n = 65) mice. Lipid and metabolite abundances differed significantly across tissue types with the most differences seen in the liver and plasma of *Trem2*^R47H^ mice at the 4-month timepoint. Cross-tissue correlation analyses revealed increased metabolic crosstalk along the liver-plasma-brain axis in *Trem2*^R47H^ mice. Plasma triacylglyceride levels were significantly lower in females compared to males regardless of genotype, and 5-methyltetrahydrofolic acid levels were elevated in the brains of animals homozygous for the *Trem2*^R47H^ variant. Together, these findings demonstrate early dyshomeostasis of the liver-plasma-brain axis of *Trem2*^R47H^ mice which impacts several key metabolic pathways involving lipids, cellular energy metabolism, and brain folate metabolism.

## 1. Introduction

Alzheimer’s disease (AD) is the most common cause of dementia worldwide (Olufunmilayo and Holsinger, 2022) and the seventh leading cause of death in the United States (Alzheimer’s Association., 2024). AD is a neurodegenerative disease characterized by extracellular amyloid β (Aβ) and intracellular neurofibrillary tangles (NFTs) of hyperphosphorylated tau (Adlard and Cummings, 2004). These neurotoxic aggregates result in the loss of cortical neurons and synapses (Moolman et al., 2004; Price and Sisodia, 1998), causing progressive impairment in memory and other cognitive function and behavior (Chen et al., 1998).

Microglia are central nervous system (CNS)-resident immune cells that phagocytose Aβ by secreting Aβ-digesting proteases (Qin et al., 2021), but the overall relation to AD pathogenesis and the effect this has on the disease course has been hotly debated. Recent studies indicate that physiological microglial function decreases amyloid pathology (Hellwig et al., 2015; Leyns et al., 2019; Parhizkar et al., 2019), while others suggest that it increases amyloid production (Rogers et al., 1999; Wegiel et al., 2000). Although these observations may seem contradictory, they may suggest that microglial activity varies over the course of AD pathophysiology, especially in relation to amyloid accumulation (Jay et al., 2017a). Considering these findings, Qin *et al.* (Qin et al., 2021) proposed that microglial activation initially exacerbates AD pathogenesis by promoting plaque seeding, but later contributes to plaque compaction and clearance, thereby ameliorating amyloid pathology in advanced stages. Alternately, microglial function may vary dynamically such that the presence of amyloid early in the course of the pathophysiological cascade may trigger an initial upregulation of microglial phagocytosis, which then plateaus, or even is exhausted as amyloid continues to accumulate (Pomilio et al., 2020).

Much of the recent work on microglial activity in AD has implicated the role of Triggering Receptor Expressed on Myeloid cells 2 (TREM2), a transmembrane receptor expressed primarily by microglia in the brain and macrophages in other organs that modulates immune responses to a variety of antigens including amyloid (Colonna, 2023; Ulland and Colonna, 2018). Located on chromosome 6p21.1, *TREM2* is a 692 base-pair gene encoding a 230 amino acid single-pass transmembrane glycoprotein (Qin et al., 2021). Physiological TREM2 signaling is vital to several microglial processes including cell activation and proliferation (Xing et al., 2015), cytokine production (Xing et al., 2015), phagocytosis and debris clearance (Gawish et al., 2015), and downregulating neurodegeneration-induced inflammation (Takahashi et al., 2005). TREM2’s extracellular domain is capable of binding damage-associated molecular pattern molecules (DAMPs) (Konishi and Kiyama, 2018; Painter et al., 2015), lipids (Tran et al., 2023; Wang et al., 2015), ApoE4 (Olufunmilayo and Holsinger, 2022), C1q (Zhong et al., 2023) and aggregated Aβ (Jiang et al., 2014; Zhao et al., 2018), all of which are implicated in the pathology of AD.

*TREM2*^*R47H*^ is a rare variant of the gene that contains a missense point mutation in the extracellular binding domain which conveys altered binding affinity (Popescu et al., 2023; Song et al., 2017; Tran et al., 2023; Wang et al., 2015). Most prevalent in Caucasian populations (Qin et al., 2021), the minor allele frequency (MAF) of the *TREM2*^*R47H*^ variant ranges from 0.12 to 0.26% in the United States, to up to 2% in some specific British populations (Jay et al., 2017b). This variant has been associated with a two- to three-fold increase in late-onset AD (LOAD) risk (Guerreiro et al., 2013; Jonsson et al., 2013), presumably through a diminished microglial response to plaques and neuronal damage. Indeed, a new mouse model of the homozygous *R47H* genotype developed by Tran *et al*. (Tran et al., 2023) demonstrates impaired microglial interactions with plaques, increased regional Aβ load, suppressed inflammatory response, and increased neuronal damage compared to wild type (WT) controls.

*TREM2* variants may also impact other important cellular processes, most notably lipid homeostasis. The major mechanism for this in the brain is likely related to microglial dysfunction, as microglia are key drivers of lipid synthesis and degradation (Shippy and Ulland, 2023). *TREM2* variants also result in increased oxidative stress and chronic inflammation which are also known to interfere with lipid synthesis and degradation (Qi et al., 2025). Lastly, TREM2 deficiency impairs *de novo* synthesis of nicotinamide adenine dinucleotide (NAD+) which is critical for the NAD-dependent deacetylase protein Sirtuin 3 (SIRT3) to regulate the mitochondrial oxidative stress response and neuroinflammation (Li et al., 2022).

Although TREM2 has been primarily studied in the context of microglial function and the CNS, its expression in peripheral myeloid cell populations suggests that the *TREM2*^*R47H*^ variant may have effects that extend beyond the brain. Notably, emerging evidence implicates liver dysfunction in the pathogenesis of AD as part of a liver-plasma-brain axis, in which impaired hepatic clearance of circulating Aβ contributes to disease pathology (Bassendine et al., 2020; Estrada et al., 2019). Given the established roles of TREM2 in immune modulation, lipid homeostasis, and metabolic regulation, we hypothesized that TREM2-associated microglial dysfunction disrupts lipid and cellular energy metabolism through a liver-plasma-brain axis, and that this effect is greatest in the presence of accumulating amyloid plaques. Thus, we examined the effects of *Trem2*^R47H^ on lipids and targeted metabolites in the liver, circulating blood, and brain using 5xFAD and *Trem2*^R47H^ mouse models to define the molecular pathways linking this variant to AD risk.

## 2. Methods

### 2.1. Mouse housing and production

All experiments involving mice were approved by the UC Irvine Institutional Animal Care and Use Committee (IACUC) and were conducted in compliance with all relevant ethical regulations for animal testing and research. All experiments involving mice comply with the Animal Research: Reporting of in Vivo Experiments (ARRIVE) guidelines.

Animals were housed in autoclaved individual ventilated cages (SuperMouse 750, Lab Products, Seaford, DE) containing autoclaved corncob bedding (Envigo 7092BK 1/8” Teklad, Placentia, CA) and two autoclaved 2” square cotton nestlets (Ancare, Bellmore, NY) plus a LifeSpan multi-level environmental enrichment platform. Tap water (acidified to pH2.5–3.0 with HCl then autoclaved) and food (LabDiet Mouse Irr 6 F; LabDiet, St. Louis, MO) were provided *ad libitum*. Cages were changed every 2 weeks with a maximum of 5 adult animals per cage. Room temperature was maintained at 72 ± 2°F, with ambient room humidity (average 40–60% RH, range 10–70%). Light cycle was 14 h light / 10 h dark, lights on at 06.30 h and off at 20.30 h.

All animals were produced by the Transgenic Mouse Facility at UC Irvine. Generation and validation of the *Trem2*^R47H NSS^ (B6(SJL)-*Trem2*^em1Aduci^, Jackson Laboratory stock #034036, hereafter referred to as *Trem2*^R47H^) allele that displays normal splicing has been described (Tran et al., 2023). C57BL/6J (Jackson Laboratory stock #000664) and 5xFAD Tg mice (B6.Cg-Tg(APPSwFlLon,PSEN1*M146L*L286V) 6799Vas/Mmjax, Jackson Laboratory Stock #34848) were purchased from Jackson Laboratory or NIH’s Mutant Mouse Resource & Research Centers (MMRRC) respectively. Experimental and control littermates were produced by natural mating or *in vitro* fertilization (IVF) procedures. Four genotypes were analyzed; (i) *Trem*2^R47H^ homozygous (referred to as *Trem2*^R47H^), (ii) 5xFAD hemizygous (referred to as 5xFAD), (iii) 5xFAD hemizygous, *Trem*2^R47H^ homozygous (referred to as 5xFAD, *Trem2*^R47H^), and (iv) WT mice. All mice were on a congenic C57BL/6J strain background.

After weaning, animals were housed together by sex, with littermates, until harvesting at either 4 or 12 months. Mice were harvested at these timepoints to represent early and advanced stages of amyloid deposition. 5xFAD mice begin to show early deposits of Aβ plaques in the cortex and hippocampus by 2 months of age, with rapid progression of amyloid deposition throughout the cortex by 4 months of age (Forner et al., 2021). By 12 months of age, 5xFAD mice typically exhibit high plaque burden in widespread regions of cortex and hippocampus, and cognitive and behavioral deficits are present. We examined the lipidome and metabolome of 180 mice in four groups: WT (n = 65), 5xFAD (n = 63), *Trem2*^R47H^ (n = 27), and 5xFAD, *Trem2*^R47H^ (n = 25). The mice were aged to either 4 or 12 months before harvesting tissues (Table 1). At the time of sacrifice, there were differences in body weight among the four genotype groups, but only at the 12-month timepoint (Figure S1). Animals were not fasted prior to tissue collection.

### 2.2. Post-mortem blood and organ harvest

Blood was drawn after cessation of respiration via a closed-chest cardiac blood draw, using a tuberculin syringe (1.0 mL) and a 25-gauge needle. Aspirated blood was immediately transferred to a 2.0 mL ethylenediaminetetraacetic acid (EDTA) tube, at a rate of approximately 1 mL/30 s, to mitigate hemolysis. The EDTA tube was inverted 10 times and placed in wet ice. EDTA blood tubes were processed within 1 hr of placement into ice. Blood components were separated via centrifugation at 1500 × g for 10 min at 20°C. Plasma was aspirated from the EDTA tube using a micropipette, and aliquoted as 100 microliter (μL) volumes into siliconized cryovials and stored at −80°C until metabolomic analysis. Prior to freezing, a 50μL aliquot of each plasma specimen was analyzed using a HemoCue^®^ Plasma/Low Hb photometer system to quantify hemoglobin present in plasma, as a determinant of hemolysis associated with the blood draw and injection into the EDTA tube. Samples with greater than 200mg/dL of hemoglobin were excluded. The small plasma volumes allowed for a single freeze-thaw cycle prior to metabolomic analysis. Plasma samples were stored at −80°C for less than 2 yr before being shipped in two shipments to the Lombardi Cancer Center Metabolomics Core Facility at Georgetown University for lipidomic and metabolomic analysis.

Liver and brain tissue were harvested immediately post-mortem. We attempted to harvest all three tissue types (liver, plasma, brain) from as many animals as possible to ensure completeness for cross-tissue comparisons. A total of 88 animals provided all three tissue types, 46 provided two tissue types, and 46 provided one tissue type, the majority (n = 40) providing plasma only (Table S1). Whole liver and hemibrains from each animal were immediately frozen in individual siliconized cryovials at −80° until delivered in a single shipment to the Lombardi Cancer Center Metabolomics Core Facility at Georgetown University for lipidomic and metabolomic assays. Upon receipt, the organs were processed following a detailed protocol (Astarita et al., 2018). Briefly, an ice-cold methanol solution containing a mixture of internal standards was added to each collection tube. Twenty mg of frozen tissue was transferred to each prepared tube, and tissue was homogenized. Chloroform, then water, were added, vortexing at each step, followed by centrifugation at 4000 ×g for 10 min at 4°C to separate aqueous and organic phases with a protein interface. The upper phase (polar metabolites) and the bottom phase (lipids) were recovered, transferred to separate vials, then dried using a vacuum evaporator then reconstituted for mass spectrometry analysis. Separate quality control (QC) pool samples were prepared from the lipid and polar metabolite phases.

### 2.3. Lipidomic and metabolic assays

Liquid Chromatography Mass Spectrometry (LC-MS) was used to quantify 967 lipids and 257 targeted polar metabolites in the liver, plasma, and brain samples. The targeted lipids comprised 16 classes: Acylcarnitines (ACs), Cholesterol Esters (CEs), Diacylglycerides (DAGs), Free Fatty Acids (FFAs), Hexosylceramides (HCERs), Lysophosphatidylcholines (LPCs), Lysophosphatidylethanolamines (LPEs), Monoacylglycerides (MAGs), Phosphatidic acids (PAs), Phosphatidylcholines (PCs), phosphatidylethanolamines (PEs), Phosphatidylglycerols (PGs), Phosphatidylinositols (PIs), Phosphatidylserines (PSs), Sphingomyelins (SMs), and Triacylglycerides (TAGs). Targeted polar metabolites were chosen to represent metabolites involved in the key metabolic pathways including oxidative phosphorylation, glycolysis/gluconeogenesis, citrate cycle (TCA cycle), single carbon, and folate metabolism, and amino acid synthesis among others (Table S2). For all mass spectrometry assays, we used established tandem MS approaches with National Institute of Standards and Technology (NIST) standards. See reference (Yuan et al., 2012) for more details.

LC-MS assays were run in two batches on the plasma samples only. A total of 60 plasma samples were run in January 2020, and the remaining 106 samples were run in January 2022. Liver and brain lipidomics and metabolomics were run in one batch in January 2022.

#### Targeted lipidomics:

Frozen 100μL samples were thawed in ice at room temperature. 25μL of plasma was aliquoted and combined with 125 μL of chilled isopropanol containing internal NIST standards and then the mixture was vortexed. The samples were vortexed again and kept on ice for 30 min, followed by incubation at −20°C for 30 min. The samples were removed from −20°C freezer and vortexed again and incubated at 4°C for 2 hr for complete protein precipitation. Samples were then centrifuged at 13,000 × g for 20 min at 4°C. Two μL of each sample was injected onto an Xbridge Amide 3.5μm, 4.6 X 100 mm column (Waters, Corp) using a SIL-30 AC auto sampler (Shimazdu, Corp.) connected with a high flow LC-30AD solvent delivery unit (Shimazdu, Corp.) and CBM-20A communication bus module (Shimazdu, Corp) online with QTRAP 5500 (AB Sciex Pte. Ltd.) operating in positive and negative ion mode. We used a binary solvent comprised of acetonitrile/water 95/5 with 10 mM ammonium acetate as solvent A and acetonitrile/water 50/50 with 10 mM ammonium acetate as solvent B for the resolution. Metabolites were resolved at 0.6 mL/min flow rate; initial gradient conditions started with 99.9% of solvent A, shifting towards 80% of solvent A over a time period of 5 min and 20% of solvent A over a time period of 3 min. Finally, we equilibrated to initial conditions (99.9% of solvent A) over a time period of 3 min using auto sampler temp at 15°C and oven temp at 35°C. Targeted lipidomic data were processed using MultiQuant 3.0.3 (AB Sciex Pte. Ltd.). Peak areas for individual lipid species were integrated and normalized to the peak area of the corresponding internal standard to correct for variability in extraction efficiency, ionization, and instrument performance. For analyses acquired in negative ion mode, 15:0–18:1(d7) phosphatidylcholine (PC) (MRM transition 811.6 → 288.3) was used as the internal standard. For analyses acquired in positive ion mode, 15:0–18:1(d7)-15:0 triacylglycerol (TAG) (MRM transition 829.8 → 570.6) was used as the internal standard. Normalized intensities were obtained by dividing the peak area of each lipid species by the peak area of the corresponding internal standard, and these normalized values were used for all downstream quantitative and statistical analyses. MS conditions were as follows: Curtain Gas = 30; CAD Gas = Medium; Ion Spray Voltage = 5.5 kV in positive mode and −4.5 kV in negative mode; Temp = 550 °C; Nebulizing Gas = 50; and Heater Gas = 60.

#### Targeted polar metabolomics:

257 polar metabolites were quantified by MS using an Ultra Performance Liquid Chromatography-Mass Spectrometry (UPLC-MS) system. The samples were resolved on a Kinetex 2.6μm 100 Å 100 × 2.1 mm (Phenomenex) column online with a triple quadrupole mass spectrometer (5500 QTRAP, AB Sciex Pte. Ltd) operating in the multiple reaction monitoring (MRM) mode. Five μL of sample was injected onto a Kinetex 2.6 μm 100 Å 100 × 2.1 mm (Phenomenex) using SIL-30 AC auto sampler (Shimazdu, Corp) connected with a high flow LC-30AD solvent delivery unit (Shimazdu, Corp) and CBM-20A communication bus module (Shimazdu, Corp) online with QTRAP 5500 (AB Sciex Pte. Ltd) operating in positive and negative ion mode. The extracted metabolites were resolved at 0.2 mL/min flow rate starting with 100% of solvent A and held for 2.1 min and moving to 5% of solvent A over 12 min and held for 1 min and equilibrating to initial conditions over 7 min using auto sampler temp 15 °C and oven temp 30 °C. We used a binary solvent comprised of water (with 0.2% formic acid) and acetonitrile (with 0.2% formic acid). Targeted metabolomic data were processed using MultiQuant 3.0.3 (AB Sciex Pte. Ltd.). Peak areas for individual metabolites were integrated and normalized to the peak area of the corresponding internal standard to correct for variability in extraction efficiency, ionization, and instrument performance. For analyses acquired in negative ion mode, 4-nitrobenzoic acid (MRM transition 166 → 122) was used as the internal standard. For analyses acquired in positive ion mode, debrisoquine (DBQ; MRM transition 176 → 115.4) was used as the internal standard. Normalized intensities were obtained by dividing the peak area of each metabolite by the peak area of the corresponding internal standard, and these normalized values were used for all downstream quantitative and statistical analyses. The sample queue was randomized, and solvent blanks were injected to assess sample carryover. Pooled QC-based robust LOESS signal correction (QC-RLSC) was applied to correct for signal drift and batch effects prior to downstream analysis (Li et al., 2017). In this approach, pooled QC samples (prepared by mixing small aliquots of all study samples) were injected regularly throughout the analytical run (here, after every six study samples) to monitor and instrumental variability over time. For each detected feature, a LOESS (locally estimated scatterplot smoothing) regression is fitted to the intensity of the QC injections as a function of injection order, and the resulting curve is used to estimate the time dependent systematic variation for that feature. The intensities of all study samples are then scaled by the fitted QC curve so that the feature’s response is effectively normalized to the QC baseline, thereby reducing non biological variance due to drift and batch effects. MS source and gas settings were set as follows: Curtain Gas = 17; CAD Gas = Medium; Ion Spray Voltage = 4.1 kV in positive mode and −4.1 kV in negative mode; Temp = 500 °C; Nebulizing Gas = 17; and Heater Gas = 25.

### 2.4. Statistical analysis

Normalized lipidomic and metabolomic data were filtered as follows. Lipids and metabolites were scanned for completeness (< limit of detection or missing data), then visualized using boxplots, and finally screened for outliers using a 3x interquartile range (IQR) fence. Outliers were replaced with a null value and any lipid or metabolite with > 33.3% null values were considered invalid and excluded from further analysis. For cells identified as null due to 1) system missing, 2) below limit of detection (LOD) or 3) outlier, we imputed a value using a K-nearest neighbors approach implemented in the R package ‘impute’ where K= 10 (Hastie et al., 2025). We then addressed potential batch effects in the plasma lipidomic and metabolomic datasets because they were run in two separate batches. We performed Principal Variance Components Analysis (PVCA) on the 106 sample by 967 lipid and the 106 sample by 257 polar metabolite matrices, showing that the plasma metabolomes were grouped together by batch. We then used the R package ‘sva’ to execute “ComBat” (Leek et al., 2012), a non-parametric empirical Bayes framework, to mitigate batch effects in the plasma datasets. A follow-up PVCA analysis showed that the plasma metabolomes no longer grouped together by batch, and thus batch effects were successfully reduced. The normalized, filtered, and batch-corrected lipid and polar metabolite abundances, from here on referred to simply as analyte abundances, were thus used as input for statistical analysis.

The abundance of each lipid and metabolite was tested for a normal distribution via Shapiro-Wilk normality test, and no abundances were normally distributed. Permutational Multivariate Analysis of Variance (PERMANOVA) was used to examine the main effects of genotype (WT; *Trem2*^R47H^; 5xFAD; and 5xFAD, *Trem2*^R47H^), sex (M, F), and age (4mo, 12mo) and the interaction of genotype and sex on the abundances of the measured lipids and polar metabolites within each age. The non-parametric PERMANOVA test was used to examine group differences based on random permutations of tests of significance rather than an F-test based on an assumption of normality. PERMANOVA was implemented using log10-transformed analyte abundances and either 9999 or 999 permutations, without False Discovery Rate (FDR) correction, using the following formulas: adonis2(select(lip_perma_df,!Name:Background)~Group2+Sex+Age,data=lip_perma_df,permutations=9999,method=“euclidean”) to examine the main effects and pairwise.adonis2(select(lip_perma_df,!Name:Background)~Group2*Sex,data=lip_perma_df,nperm=999,method=“euclidean”) to examine the interaction of genotype and sex within each age (Anderson, Braak., 2003). The difference in the number of permutations used was due to the total computational time needed. Our main comparisons of interest were isolated to address the effects of the *Trem2*^R47H^ variant and 5xFAD transgene compared to WT and critically, the effects of *Trem2*^R47H^ in the presence of amyloid plaques (i.e. in 5xFAD, *Trem2*^R47H^ mice). To address the latter question, we compared the *Trem2*^R47H^ group to the 5xFAD, *Trem2*^R47H^ group. After exploring the main effects and interactions using PERMANOVA, we performed differential abundance (DA) analysis on the analyte abundances. We required a fold change > 1.5 and Wilcoxon rank sum FDR *q* < 0.05 for identifying significantly DA lipids and metabolites, and constructed volcano plots to visualize these results using ggplot2 (Wickham, 2016). We then used MetaboAnalyst (6.0) (Pang et al., 2024) to conduct Pathway Analysis mapping to Kyoto Encyclopedia of Genes and Genomes (KEGG) *Mus musculus* pathways with all significantly DA lipids and metabolites. We created heatmaps of the top 100 DA lipids and metabolites’ log10-transformed abundances using the pheatmap R package (Kolde, 2025). For the plasma heatmaps, the color scale was manually set to an abundance range of −1.5–1.5 to prevent outliers from obscuring the observed effects. The Spearman correlations between the brain-liver, brain-plasma, and plasma-liver lipids and metabolites were generated using the “cor” function in the stats package of R. We then selected metabolites and lipids that were strongly correlated with each other (based on a Spearman’s rho value of ≥ 0.7 (Akoglu, 2018)), and combined them into a single list, one for each genotype. The genotype lists were compared and visualized with Venn diagrams using the ggVennDiagram package (Gao et al., 2021).

## 3. Results

### 3.1. Tissue-specific effects of genotype, sex, and age

**Fig. 1.**
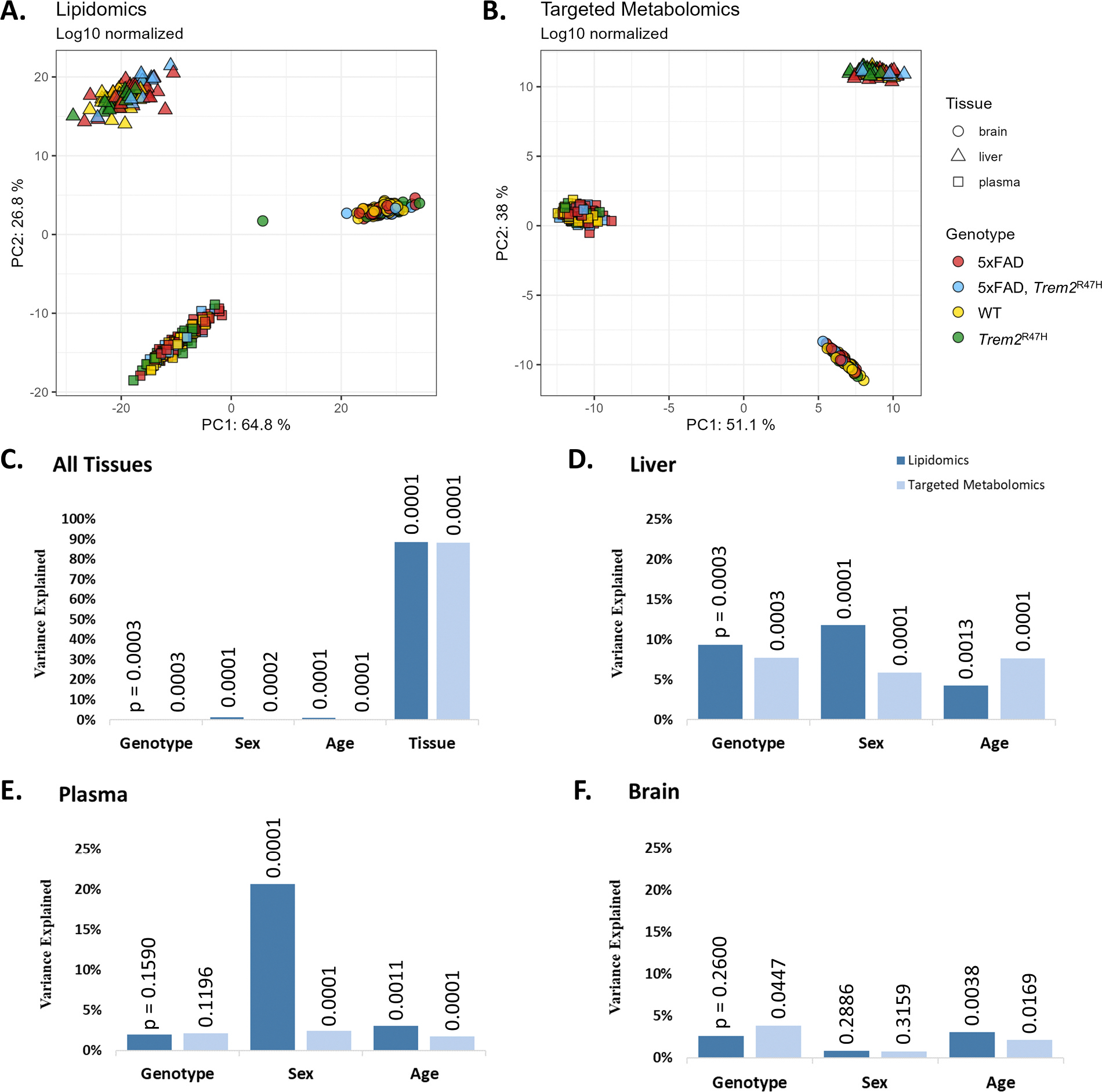
Statistical summary of the analyzed datasets. Plots of the principal variance components analysis (PVCA) for the lipidomic dataset (A) and targeted (polar) metabolite dataset (B). The plots demonstrate that principal components 1 and 2 account for most of the variance separating the three tissue types (liver, plasma, brain), and that the three tissue types have unique and non-overlapping distributions that are independent of genotype. (C) Bar plot of %R^2^ for the unstratified lipidomic (dark blue) and polar metabolite (light blue) datasets. The corresponding PERMANOVA p-values are shown above each bar. Most of the variance is attributed to tissue type (89% and 88%, respectively), and all of the factors examined are significant (*p*’s < 0.001). (D) Bar plot of %R^2^ for the liver datasets. Together, Genotype, Sex, and Age accounted for around 25% of the variance in the lipidomic dataset and 21% of the variance in the polar metabolite dataset. All three factors are significant in both datasets (*p* < 0.01 and *p* < 0.001, respectively). (E) Bar plot of %R^2^ for the plasma datasets. A large portion of the variance is explained by Sex in the lipidomic dataset (21%, *p* < 0.001), nearly ten-fold of that in the polar metabolite dataset (2.5%, *p* < 0.001). Sex and Age are significant factors in both datasets (*p* < 0.01), but Genotype is not significant in either. (F) Bar plot of %R^2^ for the brain datasets. The variance explained by each factor is relatively low. Age is a significant factor in both datasets (*p* < 0.05), and Genotype is a significant factor in the polar metabolite dataset (*p* < 0.05).

We first sought to determine if lipid and polar metabolite abundances differed between the tissues representing the liver-plasma-brain axis. PVCA for lipidomic and polar metabolite datasets revealed strong clustering of the three tissue types independent of genotype and age (Figure 1A, 1B). As expected, PERMANOVA revealed a main effect of tissue type where lipid and polar metabolite abundances were significantly different across the liver, plasma, and brain. Overall, tissue type accounted for nearly 90% of the variance in the abundances (Table 2; all *p*’s < 0.001) indicating strong separation of the three tissue types (Figure 1C). In step-down analyses, we found that both lipid and polar metabolite abundances differed by genotype, but only in the liver (*p* < 0.001; Figure 1D) and brain (*p* < 0.05; Figure 1F). Lipid and polar metabolite abundances also differed by age and sex in both the liver and plasma (*p*’s < 0.01; Figure 1D, 1E) with a striking 21% of variance accounted for by sex in the plasma and 12% in the liver lipidomic datasets (*p’s* < 0.001). Finally, brain lipid and polar metabolite abundances differed as a function of age (*p* < 0.01 and *p* < 0.05, respectively; Figure 1F). Taken together, these results show broad effects of genotype, sex, and age in the liver, and less expansive, but more powerful effects of sex and age in plasma, and more focused age effects in the brain.

### 3.2. Effects of Trem2^R47H^ and 5xFAD

We next examined the main effect of the *Trem2*^R47H^ variant on lipid and polar metabolite abundance in the liver, plasma, and brain by comparing the *Trem2*^R47H^ and WT groups. When collapsed across the two timepoints (4-and 12-months) we found only two significantly DA polar metabolites, gamma-aminobutyrate (GABA) in liver and 5-methyltetrahydrofolic acid (5-MTHF) in brain (*q*’s < 0.05; Figure S2) which were both more abundant in the *Trem2*^R47H^ group relative to the WT group.

**Fig. 2.**
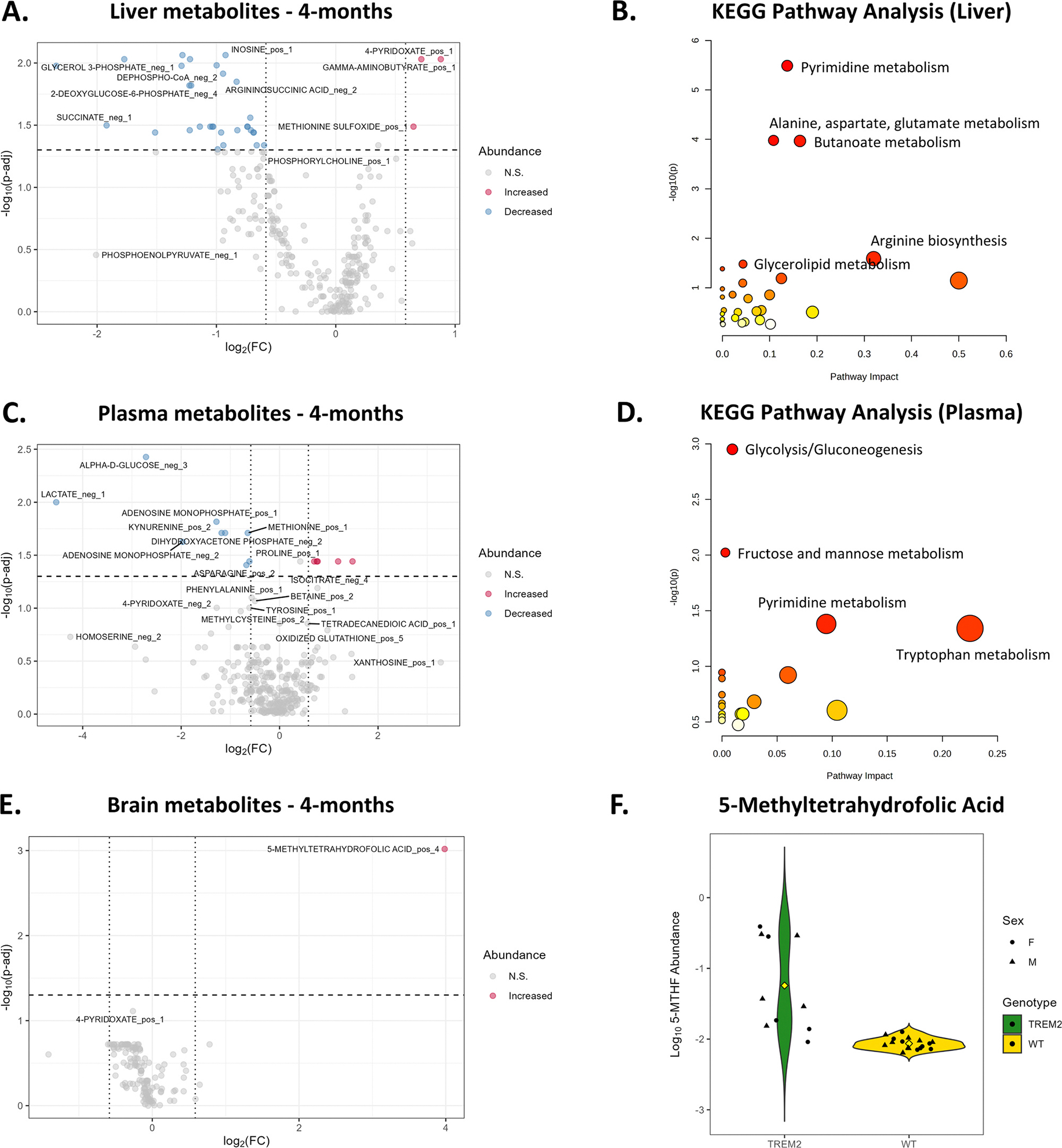
Significant effects of the *Trem2*^R47H^ variant on polar metabolites in the three tissue types. Volcano plots in panels (A, C, E) show the significantly differentially abundant (DA) polar metabolites between the WT and *Trem2*^R47H^ groups for the liver (A), plasma (C), and brain (E) at the 4-month timepoint. At 4-months, there were 33 significantly DA polar metabolites in the liver, 14 in plasma, and one in the brain. The x-axis shows the log2 fold change (FC) between genotypes. The y-axis shows the statistical significance represented as −log10 of the p-value. Each data point represents a distinct polar metabolite, with increased abundance in the *Trem2*^R47H^ group denoted in red on the right, decreased abundance in blue on the left, and non-significant metabolites in gray within the horizontal and vertical dotted lines, which indicate an FDR-adjusted p-value = 0.05 and FC cut-off values of −1.5 and 1.5, respectively. Pathway analysis maps in panels (B, D) show pathways enriched by the significantly DA polar metabolites in the liver (B) and plasma (D) at 4-months. The color of the circles representing each pathway corresponds to their significance level, and their size corresponds to their Pathway Impact score, a metric that estimates the importance of each pathway relative to the global metabolic network. (F) Violin plot of the log10 5-methyltetrahydrofolic acid abundance in the brain, between the WT and *Trem2*^R47H^ groups, at 4-months. Data points represent individual mice.

However, we found robust differences between the groups in all three tissues, particularly in liver and plasma, when examining the 4-month timepoint alone (PERMANOVA *p*’s < 0.05; Figure S3, S4, S5). At the 4-month timepoint, most of the significantly DA metabolites were less abundant in the *Trem2*^R47H^ group compared to the WT group with 30 metabolites significantly less abundant in liver and nine less abundant in plasma (*q*’s < 0.05; Figure 2A, 2C). Three metabolites were significantly more abundant in the *Trem2*^R47H^ group compared to the WT group in liver, five in plasma and one in brain (*q*’s < 0.05). Pathway analysis revealed that the DA metabolites in liver reflected changes in pyrimidine, alanine, aspartate, glutamate, and butanoate metabolism (*q*’s < 0.05; Figure 2B), as well as non-significant trends toward differences in arginine biosynthesis and glycerolipid metabolism. In plasma there were no FDR-corrected significant pathway enrichments (*q*’s > 0.05), although there was a non-significant trend for differences in glycolysis/gluconeogenesis (*q* = 0.089; Figure 2D). In addition, enrichment on pathways reflecting fructose and mannose metabolism, pyrimidine metabolism, and tryptophan metabolism met nominal (*p* < 0.05), but not FDR-corrected significance. A single polar metabolite, 5-MTHF, was more abundant in the *Trem2*^R47H^ group brain (Figure 2E, 2F). There were no significantly DA lipids between the *Trem2*^R47H^ and WT groups in liver, plasma, or brain at either the 4- or 12-month timepoints, nor were there any significantly DA polar metabolites at the 12-month timepoint.

**Fig. 3.**
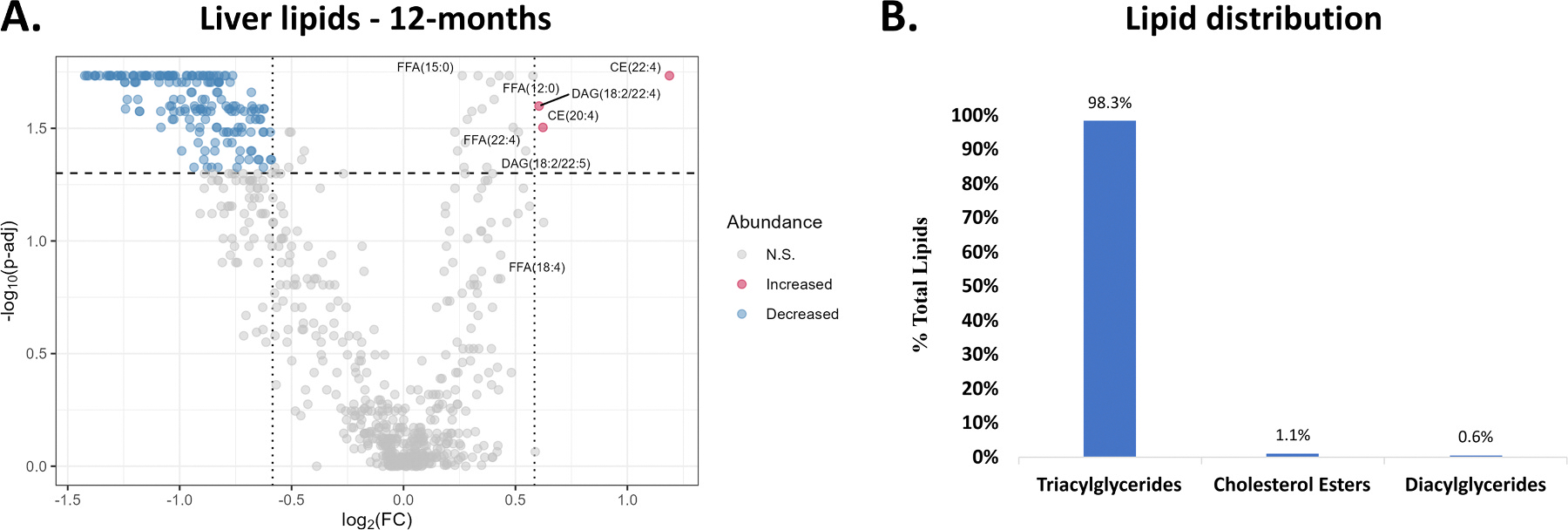
Significant effects of the 5xFAD transgene on lipids in the liver. (A) Volcano plot of the significantly differentially abundant (DA) lipids between the WT and 5xFAD groups for the liver at 12-months. (B) Bar graph of the lipid distribution. 98% of the significantly DA lipids were TAGs, all of which were in higher abundance in the liver of WT mice compared to 5xFAD mice. Across all timepoints and tissue types, this was the only comparison with significantly DA features between the WT and 5xFAD groups.

We then examined the main effect of the 5xFAD transgene on lipid and polar metabolite abundances by comparing the 5xFAD and WT groups. When collapsed across the two timepoints (4- and 12-months), and at the 4-month timepoint alone, we found no significantly DA lipids or polar metabolites between the groups in any tissue. However, there were a large number of significantly DA lipids at the 12-month timepoint in liver (PERMANOVA *p* < 0.01; Figure 3A & Figure S3). Of these 180 DA lipids, 177 (98%) were triacylglycerides (TAGs), all of which were less abundant in the 5xFAD mice compared to the WT mice (Figure 3B). There were no significantly DA lipids at the 12-month timepoint in plasma or brain and there were no DA polar metabolites at 12-months in any tissue.

Finally, we examined the differential and synergistic effects of the *Trem2*^R47H^ variant and the 5xFAD transgene on the metabolome and lipidome. To examine differential effects, we directly compared the *Trem2*^R47H^ and 5xFAD groups to each other. When collapsed across the 4- and 12-month timepoints, the only significantly DA polar metabolites are palmitic acid in plasma and 5-MTHF in brain (Figure S6A, S6B). However, we see a significant number of DA lipids in liver, again with TAGs comprising the vast majority (Figure S6C). We also examined the interaction of the Trem2^R47H^ variant and 5xFAD transgene by comparing the 5xFAD, *Trem2*^R47H^ group to the WT group. We see a significantly lower abundance of several polar metabolites in liver (Figure S6D) and a significantly higher abundance of 5-MTHF in brain of the 5xFAD, *Trem2*^R47H^ group compared to the WT group (Figure S6E). Finally, we see broad disruption of lipids, primarily lower abundance of TAGs in the 5xFAD, *Trem2*^R47H^ group in both liver and brain (Figure S6F, S6G). Comparing the *Trem2*^R47H^ and 5xFAD, *Trem2*^R47H^ groups showed DA polar metabolites and lipids in the liver only (Figure S7).

### 3.3. Effects of Trem2^R47H^ in the presence of amyloid plaques

Our main hypothesis concerns the effect of the *Trem2*^R47H^ variant on the metabolome and lipidome in the presence of amyloid plaques, to identify the potential pathways through which the *Trem2*^R47H^ variant increases AD risk. To address this question, we compared the lipid and polar metabolite abundances from the 5xFAD group, who develop amyloid plaques, to the 5xFAD, *Trem2*^R47H^ group, who have the *Trem2*^R47H^ variant in addition to amyloid plaques (Figure S8). Across both the 4- and 12-month timepoints, we found only six significantly DA polar metabolites in liver, one in plasma, and one in brain (Figure S9) and no DA lipids in any tissue. The six DA metabolites in liver showed enrichment for the glycerolipid metabolism and glycolysis/gluconeogenesis KEGG pathways at a nominal p-value (*p* < 0.05) but failed to reach FDR-corrected significance.

**Fig. 4.**
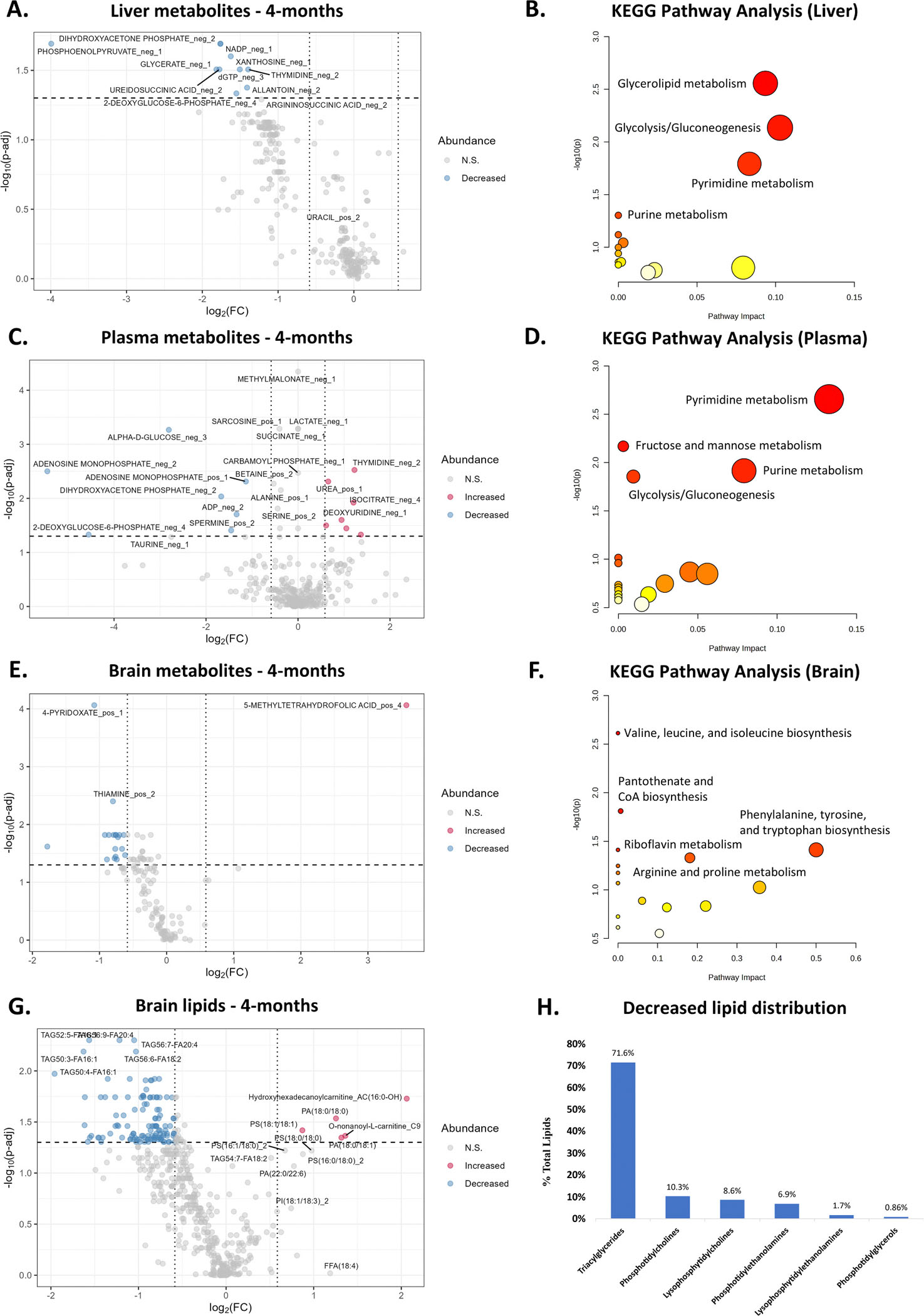
Comparison of the 5xFAD and 5xFAD, *Trem2*^R47H^ groups at 4-months. Volcano plots in panels (A, C, E, G) show the significantly differentially abundant (DA) polar metabolites between the 5xFAD and 5xFAD, *Trem2*^R47H^ groups for the liver (A), plasma (C), and brain (E), and the significantly DA lipids for the brain (G), at 4-months. There were 10 significantly DA polar metabolites in the liver, 14 in plasma, and 19 in the brain, and 121 significantly DA lipids in the brain. Pathway analysis maps in panels (B, D, F) show pathways enriched by the significantly DA polar metabolites in the liver (B), plasma (D), and brain (F) at 4-months. (H) Bar graph of the lipid distribution. 116 of the 121 significantly DA lipids had decreased abundance in the brains of 5xFAD, *Trem2*^R47H^ mice compared to 5xFAD mice, most of which were TAGs.

However, examination of the two timepoints separately revealed significantly DA polar metabolites measured in liver, plasma, and brain at the 4-month timepoint (PERMANOVA *p*’s < 0.05; Figure S3, S4, S5 & Figure 4). The ten DA polar metabolites in liver (Figure 4A) are enriched on several metabolic pathways at a nominal p-value (*p* < 0.05) including glycerolipid metabolism, glycolysis/gluconeogenesis, and pyrimidine and purine metabolism (Figure 4B), although these did not meet FDR-corrected significance. In plasma, the 14 DA polar metabolites (Figure 4C) loaded on similar metabolic pathways as in liver except for fructose and mannose metabolism replacing glycerolipid metabolism (Figure 4D). Finally, the 19 DA polar metabolites in brain (Figure 4E) loaded on amino acid biosynthetic pathways involving valine, leucine, and isoleucine, phenylalanine, tyrosine, and tryptophan (Figure 4F).

We also observed a significant difference between the groups for brain lipids at the 4-month timepoint (PERMANOVA *p* < 0.01; Figure S5) where 116 of 121 DA lipids were significantly less abundant in the 5xFAD, *Trem2*^R47H^ group compared to the 5xFAD group (Figure 4G). Most of the depleted lipids in the 5xFAD, *Trem2*^R47H^ group were TAGs (71.6%, 83/116), phosphatidylcholines (PCs) (10.3%, 12/116) or lysophosphatidylcholines (LPCs) (8.6%, 10/116) (Figure 4H).

### 3.4. Effects of genotype along the liver-plasma-brain axis

**Fig. 5.**
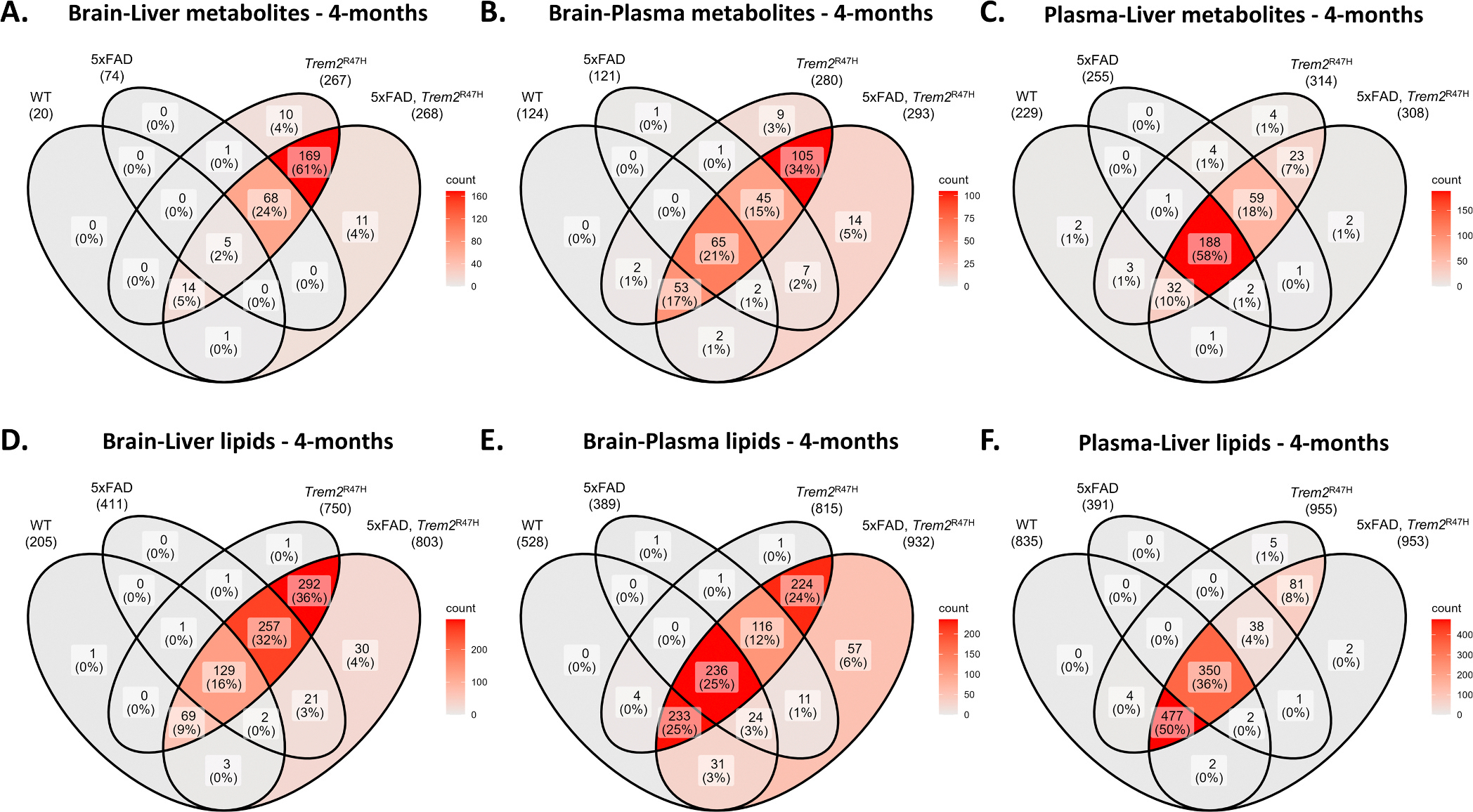
Lipid and metabolite associations along the liver-plasma-brain axis at the 4-month timepoint. Venn diagrams of strongly correlated (Spearman’s rho > 0.7) brain and liver metabolites (A), brain and plasma metabolites (B), plasma and liver metabolites (C), brain and liver lipids (D), brain and plasma lipids (E), and plasma and liver lipids (F) across the four AD genotypes at the 4-month timepoint. The number of correlated analytes is indicated in parentheses below each genotype label. Color intensity increases with the number of analytes in each section.

After investigating the effects of the *Trem2*^R47H^ variant and 5xFAD transgene on each tissue in isolation, we sought to investigate the effects of these AD genotypes along the liver-plasma-brain axis. We performed Spearman correlation analyses comparing brain-liver, brain-plasma, and plasma-liver lipids and metabolites within each genotype at both the 4-and 12-month timepoints. For each axis, we identified lipids and metabolites that were strongly correlated across tissues (Spearman’s rho > 0.7). Then, we directly compared the resulting lists of correlated analytes across genotypes, visualizing these comparisons with Venn diagrams (Figure 5 & Figure S10).

Between the brain and liver, there were more strongly correlated metabolites in mice with the *Trem2*^R47H^ variant than in mice without (Figure 5A). WT and 5xFAD mice had 20 and 74 strongly correlated metabolites across the brain and liver, respectively, whereas *Trem2*^R47H^ and 5xFAD, *Trem2*^R47H^ mice had 267 and 268 strongly correlated metabolites. Furthermore, there was substantial overlap in the specific metabolites that were correlated in the *Trem2*^R47H^ and 5xFAD, *Trem2*^R47H^ groups (61%). Between metabolites in the brain and plasma, the effect was less pronounced, but still apparent (Figure 5B). WT and 5xFAD mice had around 120 strongly correlated metabolites, whereas *Trem2*^R47H^ and 5xFAD, *Trem2*^R47H^ mice had around 280, with a 34% overlap in the correlated metabolites between the *Trem2*^R47H^ variant groups. These results suggest that mice carrying the *Trem2*^R47H^ variant, and to a lesser extent the 5xFAD transgene, have increased “crosstalk” between the brain and liver and between the brain and plasma. In contrast, the greatest overlap of correlated plasma-liver metabolites was in the section shared between all four genotype groups (58%), suggesting that AD genotype has less influence on crosstalk between the plasma and liver (Figure 5C). Similar patterns were observed for lipids (Figure 5D, 5E, 5F).

These trends also held for the 12-month timepoint (Figure S10), with the exception of the brain-plasma axis, in which the highest number of strongly correlated analytes, and the highest percentage of overlapping analytes, were found in the 5xFAD, *Trem2*^R47H^ mice alone (Figure S10B, S10E). This finding prompted an in-depth analysis of the interaction between the *Trem2*^R47H^ variant and 5xFAD transgene along the liver-plasma-brain axis, particularly between brain and plasma. At the 4-month timepoint, 14 metabolites and 57 lipids were identified in the 5xFAD, *Trem2*^R47H^ mice, that were not correlated in either 5xFAD or *Trem2*^R47H^ mice. Pathway analysis of correlated metabolites showed nominal enrichment on the pentose phosphate and glyoxylate and dicarboxylate metabolism pathways (p < 0.05; Figure S11A), and correlated lipids were largely composed of TAGs and phospholipids (Figure S11B). At the 12-month timepoint, the number of unique analytes increased substantially to 96 metabolites and 407 lipids. Correlated metabolites were significantly enriched on seven pathways, most notably the citrate cycle (TCA cycle), one carbon pool by folate, and glycolysis/gluconeogenesis (FDR *q* < 0.05; Figure S11C). Most correlated lipids were TAGs and phospholipids (Figure S11D). In light of our earlier findings, although AD genotype did not significantly alter any lipids or metabolites at the 12-month timepoint, the 5xFAD, *Trem2*^R47H^ genotype may still influence the ways in which the brain and plasma interact.

### 3.5. Effects of sex

**Fig. 6.**
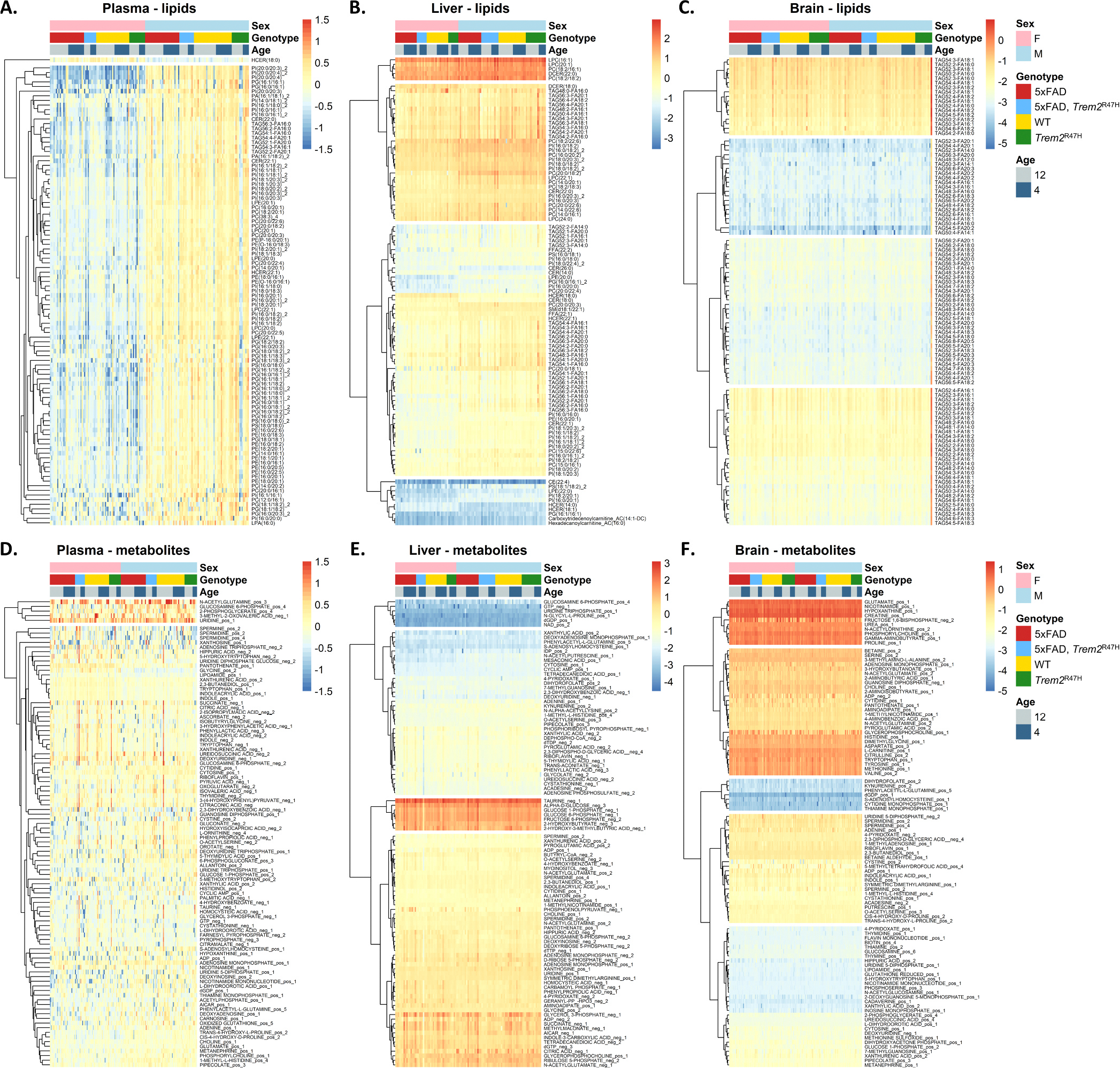
Heatmaps of sex effects. Heatmaps illustrate the log10 abundances of metabolites detected in plasma (A, D), liver (B, E), and brain (C, F). Metabolic abundances were assessed for the lipidomic (A, B, C) and targeted (polar) metabolite datasets (D, E, F) separately. Metabolites and lipids were hierarchically clustered using a dendrogram based on Euclidean distances, while samples were organized in the order of sex, genotype, and age. Only the top 100 most differentially abundant metabolites and lipids between males and females, based on log2 fold change, were shown.

Lastly, across all group comparisons, we saw a robust effect of sex on lipid and polar metabolite abundances in the liver and plasma, but not in the brain (Table 2; PERMANOVA *p*’s < 0.001; Figure 6). The effect of sex was strongest in plasma, where lipids were significantly depleted in female mice regardless of genotype or age (Figure 6A). Sex effects were also evident in 23/24 pairwise genotype comparisons (PERMANOVA *p*’s < 0.01) across both the liver and plasma (Figure S3, S4). Among these group comparisons, sex accounted for an average of 16.5% of the variance between the groups in the liver (range 12%–22%) and 25.9% of the variance between the groups in the plasma (range 16%–40%). (Figure S3, S4). The effect of sex on lipid abundances in the brain was observed only at the 4-month timepoint (PERMANOVA *p* < 0.05), suggesting early effects on brain lipid abundances (Figure S5).

## 4. Discussion

In this study we investigated the effects of the *TREM2*^*R47H*^ AD risk-variant on the lipidome and metabolome in the aging mouse. We examined the liver, peripheral plasma, and brain of WT, *Trem2*^R47H^, 5xFAD, and 5xFAD, *Trem2*^R47H^ mice at two timepoints (4- and 12-months). We were interested in the main effects of the *Trem2*^R47H^ variant and the 5xFAD transgene independently (each compared to WT), the differential and synergistic effects of the *Trem2*^R47H^ variant and 5xFAD transgene (*Trem2*^R47H^ compared to 5xFAD, and WT compared to 5xFAD, *Trem2*^R47H^ respectively), and the effect of *Trem2*^R47H^ in the presence of amyloid plaques (5xFAD compared to 5xFAD, *Trem2*^R47H^). Our results reveal early and robust effects of *Trem2*^R47H^ on the lipidome and metabolome affecting all three tissue types, but strongest in liver and mostly observed at the 4-month timepoint. Metabolic pathways implicated amino acid and glycerolipid metabolism among others, while lipid differences were greatest in TAGs and glycerophospholipids including PCs and LPCs. We also observed strong effects of biological sex on the lipidome, especially in plasma, that was independent of genotype or age. Finally, both groups with the *Trem2*^R47H^ variant showed elevated 5-MTHF abundance in brain.

### 4.1. The timeline of Trem2^R47H^ mediated effects is early

TREM2 is expressed in the CNS, predominantly in microglia, where it is required for transition to the disease-activated microglia (DAM) profile for phagocytosis, protecting neuronal tissue, and increasing microglial metabolic capacity (Ulland and Colonna, 2018). With respect to AD, the *Trem2*^R47H^ variant alters the binding affinity of TREM2 to brain cell membrane phospholipids and amyloid plaques, and may therefore confer decreased metabolic capacity in the face of brain amyloid pathology (Song et al., 2017). Thus, our main analysis concerned the lipidomic and metabolomic effects of the *Trem2*^R47H^ variant in the presence of amyloid-related AD pathology (5xFAD, *Trem2*^R47H^ vs 5xFAD). Here, we observed pronounced effects at the 4-month timepoint when nearly all 5xFAD mice show compact, extracellular fibrillar amyloid in brain (Forner et al., 2021), yet found no differentially abundant lipids or metabolites in any tissue at the 12-month timepoint. Our finding of altered lipids and metabolites supports the idea that altered microglial activity (vis-à-vis *Trem2*^R47H^ expression) occurs early in the time course of AD pathophysiology. This early change is consistent with our previous report in 5xFAD, *Trem2*^R47H^ mice showing reduced microglia size and number, and impaired microglia-plaque interaction at the 4-month timepoint, but not at 12-months (Tran et al., 2023).

Lipids are essential for microglial structure and function and are also critical for meeting energy needs under homeostatic stress. Changes in microglial phospholipid composition can impact membrane integrity, affecting microglial morphology and functions such as autophagy (Fourgeaud et al., 2016). Additionally, alterations in lipid raft dynamics may influence microglial signal transduction and receptor clustering, modulating their response to various stimuli, including amyloid (Di Paolo and Kim, 2011). Perhaps more importantly, lipids, especially TAGs are a critical source of cellular energy to support the immediate and high energy demands of activated microglia in the presence of accumulating amyloid (Yassine et al., 2023) and this may be impacted by lipid-related factors such as apolipoprotein E (*APOE*) genotype (Haney et al., 2024). It is perhaps unsurprising then, that the metabolic pathways implicated in our study included glycerolipid, predominantly TAG metabolism and glycolysis/gluconeogenesis. These findings support the idea of an early change in cellular energy source from glucose metabolism to fatty acid beta-oxidation, perhaps related to increased microglial energy demands in the presence of accumulating amyloid. In comparison, the lack of significant lipid and metabolic differences between the 5xFAD, Trem2^R47H^ vs 5xFAD groups at 12-months may represent failure of this compensatory mechanism.

### 4.2. The Trem2^R47H^ variant has prominent effects beyond the CNS

As a risk variant for AD, *TREM2*^*R47H*^ has been primarily studied in the context of the CNS, but TREM2 is expressed in all myeloid cells, and therefore also plays a significant role in immune function outside the brain. This is notable in the liver, where TREM2 is expressed in non-parenchymal Kupffer cells, hepatic stellate cells and endothelial cells (Chen et al., 2008), and modulates toll-like-receptor induced cytokine production (Esparza-Baquer et al., 2021; Perugorria et al., 2019). In several hepatic carcinogenesis and infection models, TREM2 has been shown to have protective effects on liver function through reducing inflammation and reactive oxygen species generation and increasing growth factor production (Goncalves et al., 2013; Krammer et al., 2023).

Our findings of prominent and potentially disruptive effects of the *Trem2*^R47H^ variant in the liver and peripheral plasma are consistent with the known role of TREM2 signaling in tissue-specific macrophage responses to tissue-specific lipid homeostasis, and suggest that the mechanisms behind the increase in AD risk may extend beyond the CNS. Furthermore, our analysis of the liver-plasma-brain axis revealed pronounced effects of the *Trem2*^R47H^ variant on cross-tissue metabolic associations, particularly between the brain and liver. Similarly, brain-plasma associations were increased in mice with the *Trem2*^R47H^ variant, but were most prominent in 5xFAD, *Trem2*^R47H^ mice at the 12-month timepoint. This suggests that the interaction between amyloid pathology and the *Trem2*^R47H^ variant increases crosstalk between tissues and may be indicative of blood-brain barrier dysfunction and the disruption of correlated metabolic pathways involved in cellular energy metabolism. Lastly, the effect of *the Trem2*^R47H^ variant alone (*Trem2*^R47H^ vs WT) and in the presence of amyloid plaques (5xFAD, *Trem2*^R47H^ vs 5xFAD) was substantial and suggests a key effect of the *Trem2*^R47H^ variant above and beyond that in the presence of AD pathology associated with 5xFAD.

### 4.3. Sex has prominent effects on the lipidome

We found that 620 of the 967 plasma lipids in our targeted panel were significantly altered in female mice compared to males, across all ages and genotypes, accounting for a profound 21% of the variance in abundance (PERMANOVA *p* < 0.001) (Table 2). Among these, only five were upregulated, including specific sphingomyelins (SMs), hydroxyceramides (HCERs), and dimethylnonanoyl carnitine. The remaining 359 downregulated lipids in female mice were mostly composed of TAGs (22.4%, 138/615), phosphatidylethanolamines (PEs) (19.8%, 122/615), and PCs (16.1%, 99/615). In humans, the plasma lipidome is known to be substantially different between males and females, but the directionality of these differences depends on many factors including age, menopausal status, and obesity (Palmisano et al., 2018). Many studies have demonstrated lower plasma triglyceride levels in premenopausal women compared to men (e.g., (Mikkelsen et al., 2023)) and this is attributed to enhanced clearance of triglyceride-rich lipoproteins from the bloodstream (Wang et al., 2011).

How these differences are related to the incidence of AD is not entirely clear, but it is well known that women are disproportionately affected by AD, by some estimates accounting for nearly 70% of diagnoses (Fisher et al., 2018). Estrogen production abruptly decreases post-menopause, and this decrease is associated with lower levels of anti-inflammatory long-chain polyunsaturated fatty acids (LC-PUFAs) and high-density lipoprotein cholesterol (HDL-C), and higher levels of triglycerides (Morselli et al., 2018), which have been linked to cognitive decline (Zhou et al., 2023). Low estrogen levels can also cause a decline in the expression of nerve growth factor (NGF) (Priyanka et al., 2013) and brain-derived neurotrophic factor (BDNF) (Konishi et al., 2020), which are neuroprotective.

### 4.4. The Trem2^R47H^ variant and folate metabolism

**Fig. 7.**
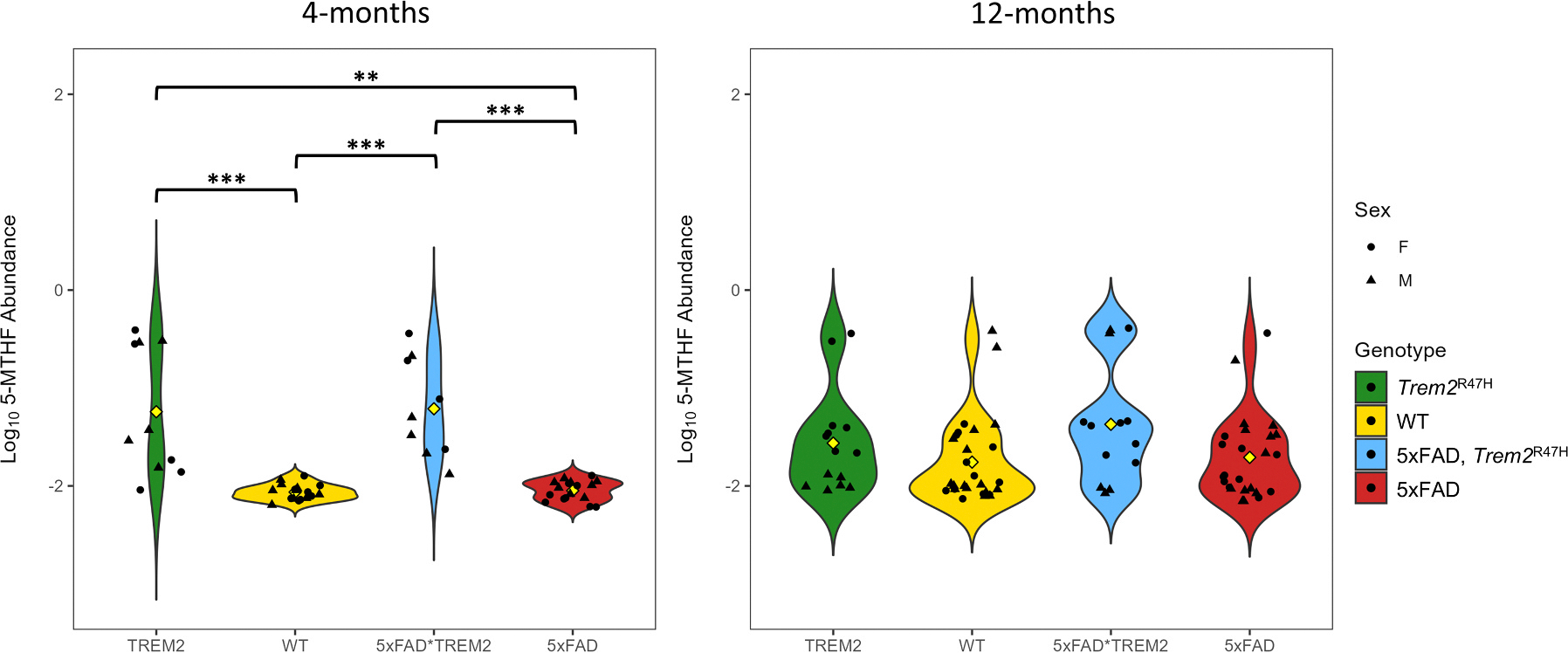
5-methyltetrahydrofolic acid abundances. Violin plot of the log10 5-methyltetrahydrofolic acid abundance in the brain, across all groups, at 4-and 12-months. Data points represent individual mice. The number of asterisks represents the significance level (Kruskal-Wallis χ^2^ = 32.924, df = 3, p = 3.342e-07; post-hoc Dunn’s test with Bonferroni correction: p < = 0.001(***), p < = 0.01 (**), and p < = 0.05 (*)). Groups with the *Trem2*^R47H^ variant showed higher abundance of 5-methyltetrahydrofolic acid relative to WT and 5xFAD control groups, but only at 4-months.

While there were very few significantly DA polar metabolites in the brain overall, we found that 5-MTHF abundance was significantly altered across most comparisons at the 4-month timepoint. To further investigate this observation, we directly compared 5-MTHF levels across all genotypes and timepoints ( Figure 7). At the 4-month timepoint, *Trem2*^R47H^ mice had significantly higher 5-MTHF abundance than mice without the variant, regardless of the presence or absence of amyloid plaques. At the 12-month timepoint, 5-MTHF abundance was equally variable between genotypes. 5-MTHF is an active form of folate (vitamin B9), functioning as a methyl donor in many downstream metabolic reactions including the metabolism of serine, glycine, and histidine, and the synthesis of several DNA precursor molecules and neurotransmitters (Scaglione and Panzavolta, 2014).

Folate deficiency has been associated with LOAD, due in part to impaired folate-mediated one carbon metabolism (Zhang et al., 2021). Moreover, folic acid supplementation has been found to be protective against the disease (Zhang et al., 2021) and has been used in several clinical trials to date, both by itself and as part of a regimen with other B-vitamins, each with promising results (Chen et al., 2021; Chen et al., 2016; Douaud et al., 2013). Given this established relationship between folate and LOAD, the early accumulation of 5-MTHF in the brains of mice carrying the *Trem2*^R47H^ variant could potentially contribute to the pathogenesis of AD. We also observed a significant dysregulation of 5-MTHF methyl acceptors methionine, histidine, and thiamine, and the monoamine neurotransmitter precursor phenylalanine, in the 4-month--old brains of 5xFAD, *Trem2*^R47H^ mice compared to 5xFAD mice (Figure 4E). These findings suggest that the *Trem2*^R47H^ variant, in the context of amyloid pathology, may exacerbate AD risk by altering brain folate metabolism and 5-MTHF-associated amino acid pathways. Additional research is needed to understand the mechanism underlying the observed 5-MTHF alteration.

## 5. Conclusion

In this study, we find evidence of broad metabolic and lipidomic alterations associated with homozygosity for the *Trem2*^R47H^ variant in the mouse. This partial loss-of-function variant was associated with depletion of lipids, primarily TAGs in the brain, when in the presence of amyloid. We interpret these findings to reflect microglial-driven, cellular energy dyshomeostasis which occurs early in the AD pathophysiological cascade. The *Trem2*^R47H^ variant was also associated with alterations of folate metabolism and more targeted studies of folate metabolism in mouse models are needed. Although bulk lipidomics and metabolomics lack the resolution to capture region- or cell type-specific alterations in the brain, the strength of the observed signals support the relevance of our findings. Future studies should address the role of lipid and cellular energy alterations to better understand these metabolic drivers and consequences of AD pathology.

## Supplementary Material

SUPP MATERIAL

Supporting information

This article contains supporting information.

## Figures and Tables

**Table 1:** Sample sizes by genotype, age, and tissue.

Genotype	4 months(females/males)				12 months(females/males)			
	Total # Mice	Liver	Plasma	Cortex	Total # Mice	Liver	Plasma	Cortex

Wild Type	27(13/14)	17(8/9)	25(12/13)	17(8/9)	38(17/21)	17(7/10)	33(15/18)	28(12/16)
5xFAD	28(14/14)	18(9/9)	26(13/13)	17(9/8)	35(17/18)	13(6/7)	30(15/15)	25(12/13)
*Trem2* ^R47H^	10(5/5)	10(5/5)	10(5/5)	10(5/5)	17(8/9)	11(2/9)	17(8/9)	14(8/6)
5xFAD, *Trem2*^R47H^	10(5/5)	10(5/5)	10(5/5)	9(4/5)	15(8/7)	9(2/7)	13(6/7)	13(8/5)
Totals	75(37/38)	55(27/28)	71(35/36)	53(26/27)	105(50/55)	50(17/33)	93(44/49)	80(40/40)

*Note.* Not all animals provided all three tissue types, so each cell may not equal the total number of animals providing samples.

**Table 2:** PERMANOVA of Main Effects.

Factor	All Tissues									
	Lipidomics					Targeted Metabolomics				
	df	SS	%R2	F-val	p-val	df	SS	%R2	F-val	p-val

Genotype	**3**	**9271**	**0.3%**	**4.933**	**0.0003**	**3**	**2348**	**0.3%**	**3.869**	**0.0003**
Sex	**1**	**31721**	**1.2%**	**50.63**	**0.0001**	**1**	**1941**	**0.3%**	**9.595**	**0.0002**
Age	**1**	**22697**	**0.8%**	**36.23**	**0.0001**	**1**	**3348**	**0.5%**	**16.55**	**0.0001**
Tissue	**2**	**2401077**	**89%**	**1916**	**0.0001**	**2**	**650084**	**88%**	**1607**	**0.0001**
Residual	392	245581	9.1%	na	na	394	79713	11%	na	na
	Plasma									
	Lipidomics					Targeted Metabolomics				
	df	SS	%R2	F-val	p-val	df	SS	%R2	F-val	p-val
Genotype	3	3485	2.0%	1.411	0.1590	3	1238	2.1%	1.186	0.1196
Sex	**1**	**35985**	**21%**	**43.69**	**0.0001**	**1**	**1440**	**2.5%**	**4.139**	**0.0001**
Age	**1**	**5283**	**3.0%**	**6.414**	**0.0011**	**1**	**1006**	**1.7%**	**2.892**	**0.0001**
Tissue	na	na	na	na	na	na	na	na	na	na
Residual	157	129319	74%	na	na	158	54965	94%	na	na
	Brain									
	Lipidomics					Targeted Metabolomics				
	df	SS	%R2	F-val	p-val	df	SS	%R2	F-val	p-val
Genotype	3	1224	2.6%	1.191	0.2680	**3**	**249.8**	**3.9%**	**1.762**	**0.0447**
Sex	1	401	0.9%	1.171	0.2886	1	50.6	0.8%	1.071	0.3159
Age	**1**	**1421**	**3.1%**	**4.146**	**0.0038**	**1**	**140.1**	**2.2%**	**2.965**	**0.0169**
Tissue	na	na	na	na	na	na	na	na	na	na
Residual	126	43117	93%	na	na	127	6001.0	93%	na	na
	Liver									
	Lipidomics					Targeted Metabolomics				
	df	SS	%R2	F-val	p-val	df	SS	%R2	F-val	p-val
Genotype	**3**	**5442**	**9.4%**	**4.142**	**0.0003**	**3**	**1339.6**	**7.7%**	**3.247**	**0.0003**
Sex	**1**	**6847**	**12%**	**15.63**	**0.0001**	**1**	**1021.2**	**5.9%**	**7.425**	**0.0001**
Age	**1**	**2472**	**4.3%**	**5.645**	**0.0013**	**1**	**1324.3**	**7.7%**	**9.630**	**0.0001**
Tissue	na	na	na	na	na	na	na	na	na	na
Residual	99	43358	75%	na	na	99	13614.7	79%	na	na

## Data Availability

The datasets generated and/or analyzed during the current study are publicly available via Harvard Dataverse (https://doi.org/10.7910/DVN/XZZCDE). An identical version of these materials, including analysis code, is also available at GitHub (https://github.com/GinaFaraci/TREM2-Metabolomics).

## Appendix A. Supporting information

Supplementary data associated with this article can be found in the online version at doi:10.1016/j.neurobiolaging.2026.04.007.
