# Supplementary material for: The TREM2 R47H variant is associated with liver-plasma-brain axis dyshomeostasis in the 5xFAD mouse model of Alzheimer’s disease
Source: Neurobiol Aging. Author manuscript; Available in PMC 2026 Jun 9. (PMC13249471; doi:10.1016/j.neurobiolaging.2026.04.007)
Supplement: SUPP MATERIAL [file NIHMS2176619-supplement-SUPP_MATERIAL.docx]

**The TREM2 R47H variant is associated with liver-plasma-brain axis dyshomeostasis in the 5xFAD mouse model of Alzheimer’s disease**

Gina Faraci^*^, Benjamin Goodfriend, Joseph Bishop, Michael Vu, Julio Avelar-Barragan, Sage JB Dunham, Jason A Rothman, Katrine L Whiteson, Amrita K Cheema, Giedre Milinkeviciute, Andrea J Tenner, Frank M LaFerla, Grant R MacGregor, Kim N Green, and Mark Mapstone^*^

**Supporting information included:**

1. Table S1. Tissues harvested from each animal.
2. Table S2. Metabolic pathways represented by targeted polar metabolites.
3. Figure S1. Mouse weight analysis.
4. Figure S2. Comparison of the WT and *Trem2*^R47H^ groups across the 4- and 12-month timepoints.
5. Figure S3. PERMANOVA results for liver tissue.
6. Figure S4. PERMANOVA results for plasma.
7. Figure S5. PERMANOVA results for brain tissue.
8. Figure S6. Differential and synergistic effects of *Trem2*^R47H^ variant and 5xFAD transgene.
9. Figure S7. Comparison of the *Trem2*^R47H^ and 5xFAD, *Trem2*^R47H^ groups across the 4- and 12-month timepoints.
10. Figure S8. Heatmaps of differences between 5xFAD and 5xFAD, Trem2R47H groups for genotype, sex, and age.
11. Figure S9. Comparison of the 5xFAD and 5xFAD, *Trem2*^R47H^ groups across the 4- and 12-month timepoints.
12. Figure S10. Lipid and metabolite associations along the liver-plasma-brain axis at the 12-month timepoint.
13. Figure S11. Synergistic effects of *Trem2*^R47H^ variant and 5xFAD transgene along the plasma-brain axis.

**Table S1.**

*Tissues harvested from each animal.*

| Genotype | Matrix | | | | | | | |
| --- | --- | --- | --- | --- | --- | --- | --- | --- |
|  | Total  (female/  male) | Liver only  (female/  male) | Plasma only  (female/  male) | Cortex only  (female/  male) | Plasma/  Cortex  (female/  male) | Liver/  Plasma  (female/  male) | Liver/  Cortex  (female/  male) | Liver/  Plasma/  Cortex  (female/  male) |
| Wild Type | 65  (30/35) | 0 | 20  (10/10) | 3  (0/3) | 8  (5/3) | 0 | 4  (3/1) | 30  (12/18) |
| 5xFAD | 63  (31/32) | 0 | 20  (10/10) | 3  (1/2) | 9  (5/4) | 1  (0/1) | 4  (2/2) | 26  (13/13) |
| *Trem2*^R47H^ | 27  (13/14) | 0 | 0 | 0 | 6  (6/0) | 3  (0/3) | 0 | 18  (7/11) |
| 5xFAD, *Trem2*^R47H^ | 25  (13/12) | 0 | 0 | 0 | 6  (6/0) | 3  (1/2) | 2  (0/2) | 14  (6/8) |
| Totals | 180  (87/93) | 0 | 40  (20/20) | 6  (1/5) | 29  (22/7) | 7  (1/6) | 10  (5/5) | 88  (38/50) |

**Table S2.**

*Metabolic pathways represented by targeted polar metabolites.*

| Metabolic pathway (number of metabolites) |
| --- |
| Citric Acid Cycle (11) |
| Diabetic cardiomyopathy (1) |
| Biosynthesis of cofactors (1) |
| Pentose phosphate pathway (6) |
| Sugar and Amino sugar metabolism (4) |
| Vitamin metabolism and biosynthesis (7) |
| Tryptophan metabolism (4) |
| Phenylalanine metabolism (4) |
| Lipoic acid metabolism (1) |
| Oxidative phosphorylation (3) |
| Nicotinate and nicotinamide metabolism (2) |
| Fatty acid metabolism (3) |
| Purine metabolism (22) |
| Amino acids synthesis, metabolism, and degradation (47) |
| Glycolysis, Gluconeogenesis and Pyruvate metabolism (10) |
| Single carbon metabolism and folate metabolism (16) |
| Pyrimidine biosynthesis (12) |
| Urea cycle (10) |
| Others (93) |

**
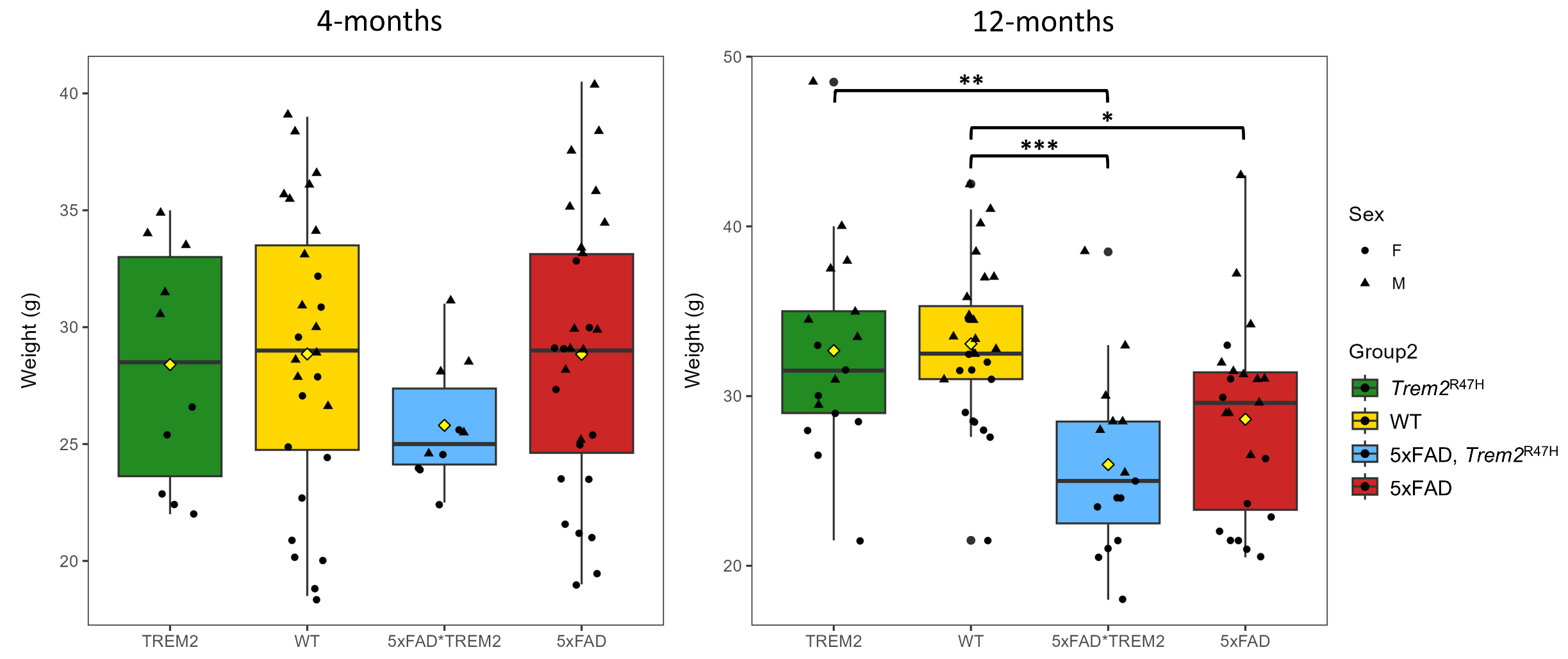
**

**Figure S1. Mouse weight analysis.** Box plot of mouse weight (in grams) across all genotype groups, at 4-and 12-months. Data points represent individual mice. The number of asterisks represents the significance level (One-way ANOVA and post-hoc Tukey HSD test with Bonferroni correction: p <= 0.001(***), p <= 0.01 (**), and p <= 0.05 (*)).

**
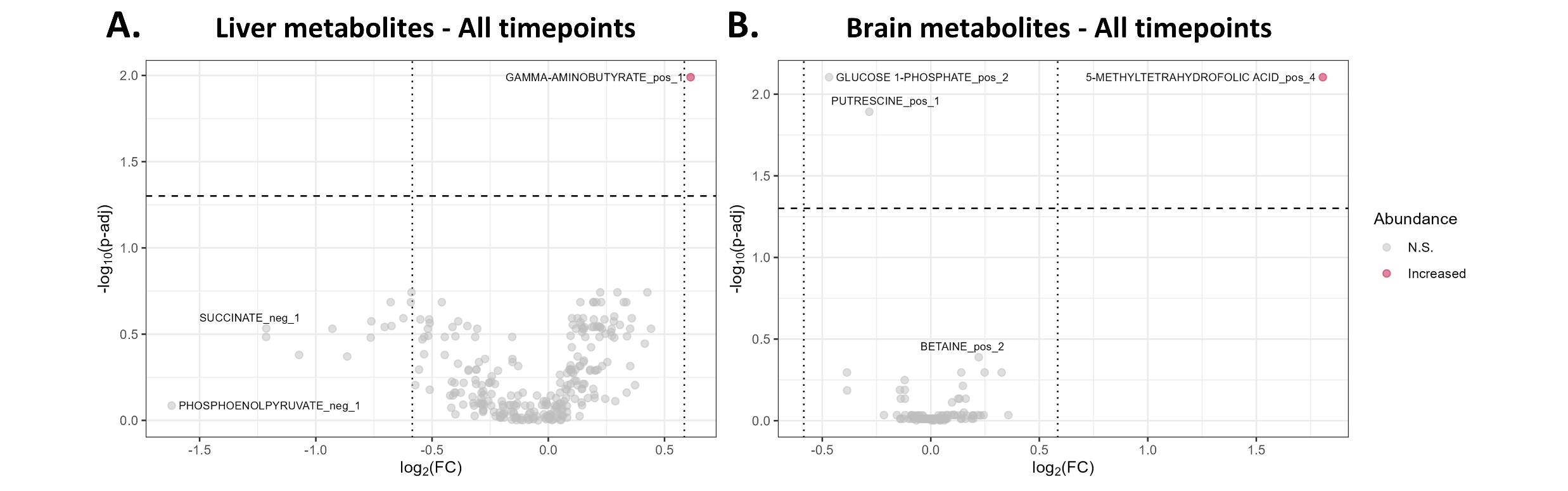
**

**Figure S2. Comparison of the WT and *Trem2*^R47H^ groups across the 4- and 12-month timepoints.** Volcano plots of the significantly differentially abundant (DA) polar metabolites between the WT and *Trem2*^R47H^ groups for the liver **(A)** and brain **(B)** across the 4- and 12-month timepoints. There was one significantly DA polar metabolite in the liver and one in the brain.

**
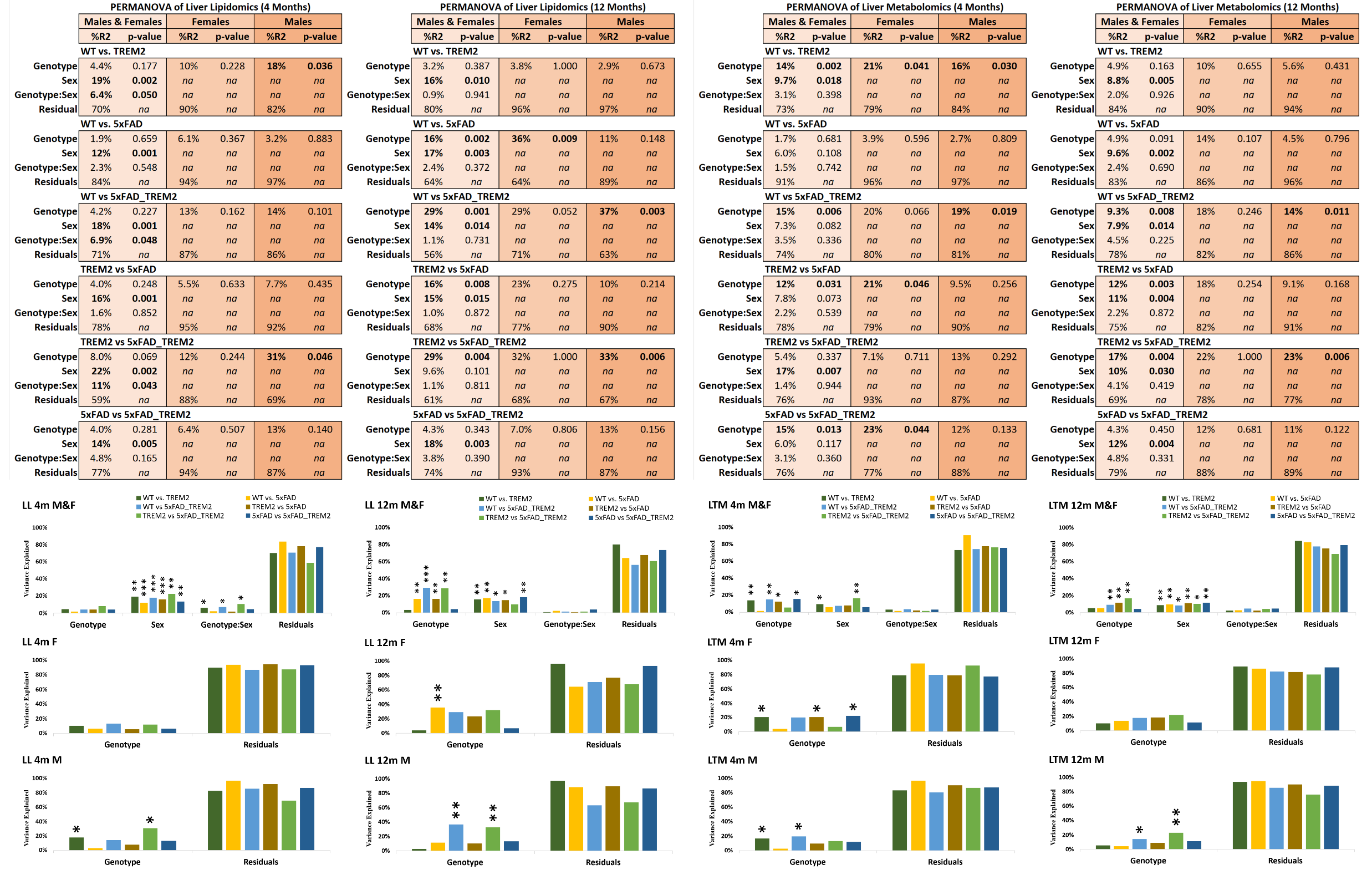
**

**Figure S3. PERMANOVA results for liver tissue.** Tables of PERMANOVA outputs (%R2 and p-value) for the liver lipidomic and polar metabolite datasets, stratified by Genotype (six pairwise comparisons between WT, *Trem2*^R47H^, 5xFAD, and 5xFAD, *Trem2*^R47H^ mice), Age (4-month and 12-month) and Sex (M&F, F, and M). The %R^2^ value represents the percentage of variance explained by each factor (Genotype and Sex). Significant effects are bolded. Below each table are the corresponding bar plots of %R^2^. Each bar represents a separate pairwise comparison between the four mouse genotype groups: WT vs. *Trem2*^R47H^ (dark green), WT vs. 5xFAD (yellow), WT vs. 5xFAD, *Trem2*^R47H^ (light blue), *Trem2*^R47H^ vs. 5xFAD (brown), *Trem2*^R47H^ vs. 5xFAD, *Trem2*^R47H^ (light green), and 5xFAD vs. 5xFAD, *Trem2*^R47H^ (dark blue). If the PERMANOVA p-value was significant for a specific comparison, an asterisk is shown above that bar. The number of asterisks represents the significance level (p = 0.001 (***), p <= 0.01 (**), and p <= 0.05 (*)). The variance and significance levels of genotype on each comparison generally increased from 4-months to 12-months in the lipidomic dataset, while the effect of sex generally decreased. In the polar metabolite dataset, the variance and significance levels of genotype on each comparison increased for some and decreased for others, while the effect of sex generally increased.


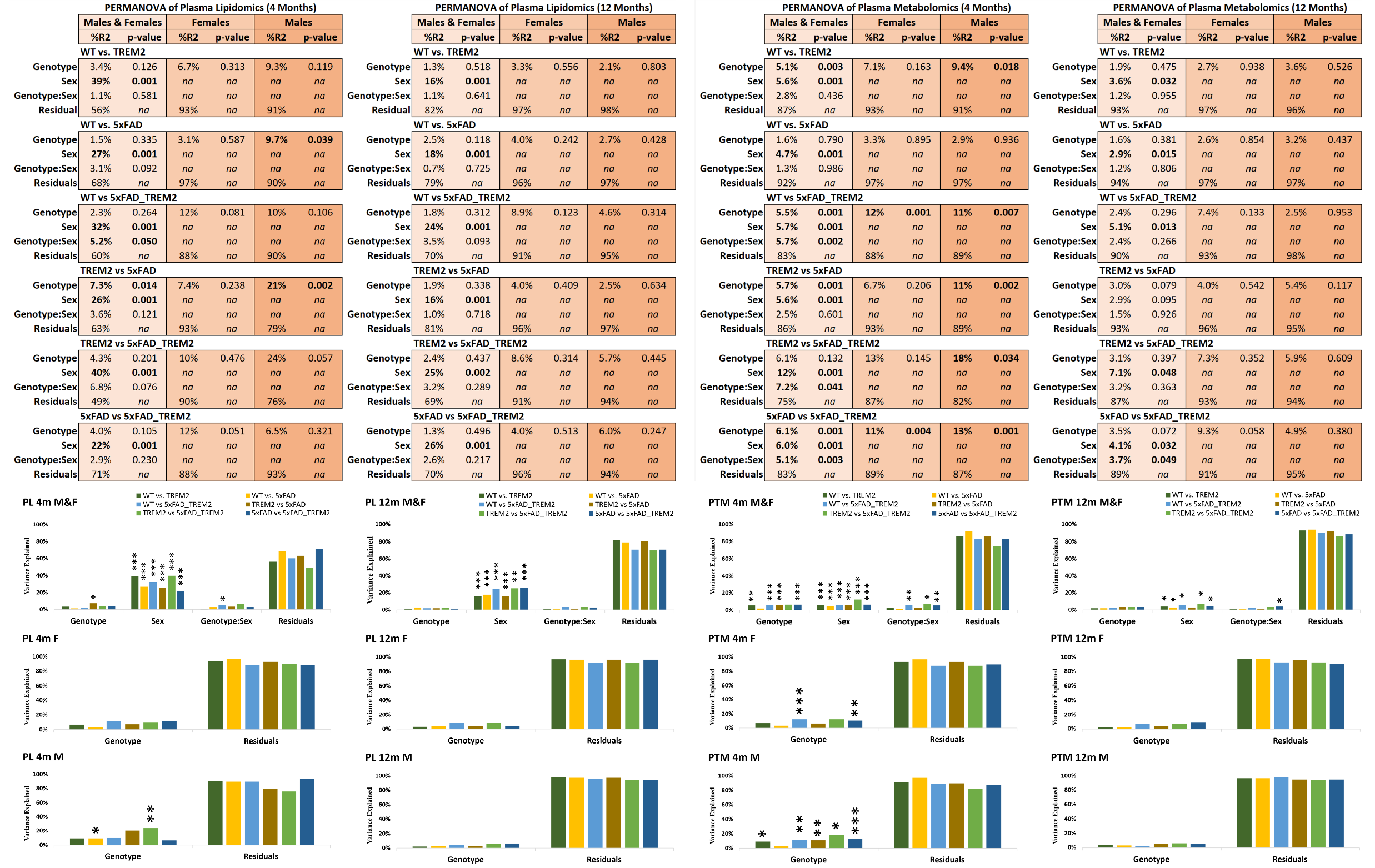


**Figure S4.** **PERMANOVA results for plasma.** Tables of PERMANOVA outputs (%R2 and p-value) for the plasma lipidomic and polar metabolite datasets, stratified by Genotype (six pairwise comparisons between WT, *Trem2*^R47H^, 5xFAD, and 5xFAD, *Trem2*^R47H^ mice), Age (4-month and 12-month) and Sex (M&F, F, and M). The %R^2^ value represents the percentage of variance explained by each factor (Genotype and Sex). Significant effects are bolded. Below each table are the corresponding bar plots of %R^2^. Each bar represents a separate pairwise comparison between the four mouse genotype groups: WT vs. *Trem2*^R47H^ (dark green), WT vs. 5xFAD (yellow), WT vs. 5xFAD, *Trem2*^R47H^ (light blue), *Trem2*^R47H^ vs. 5xFAD (brown), *Trem2*^R47H^ vs. 5xFAD, *Trem2*^R47H^ (light green), and 5xFAD vs. 5xFAD, *Trem2*^R47H^ (dark blue). If the PERMANOVA p-value was significant for a specific comparison, an asterisk is shown above that bar. The number of asterisks represents the significance level (p = 0.001 (***), p <= 0.01 (**), and p <= 0.05 (*)). In general, the variance and significance levels of each comparison decreased from 4-months to 12-months, with more significant comparisons in the 4-month male mice vs. the 4-month female mice, across both datasets.

**
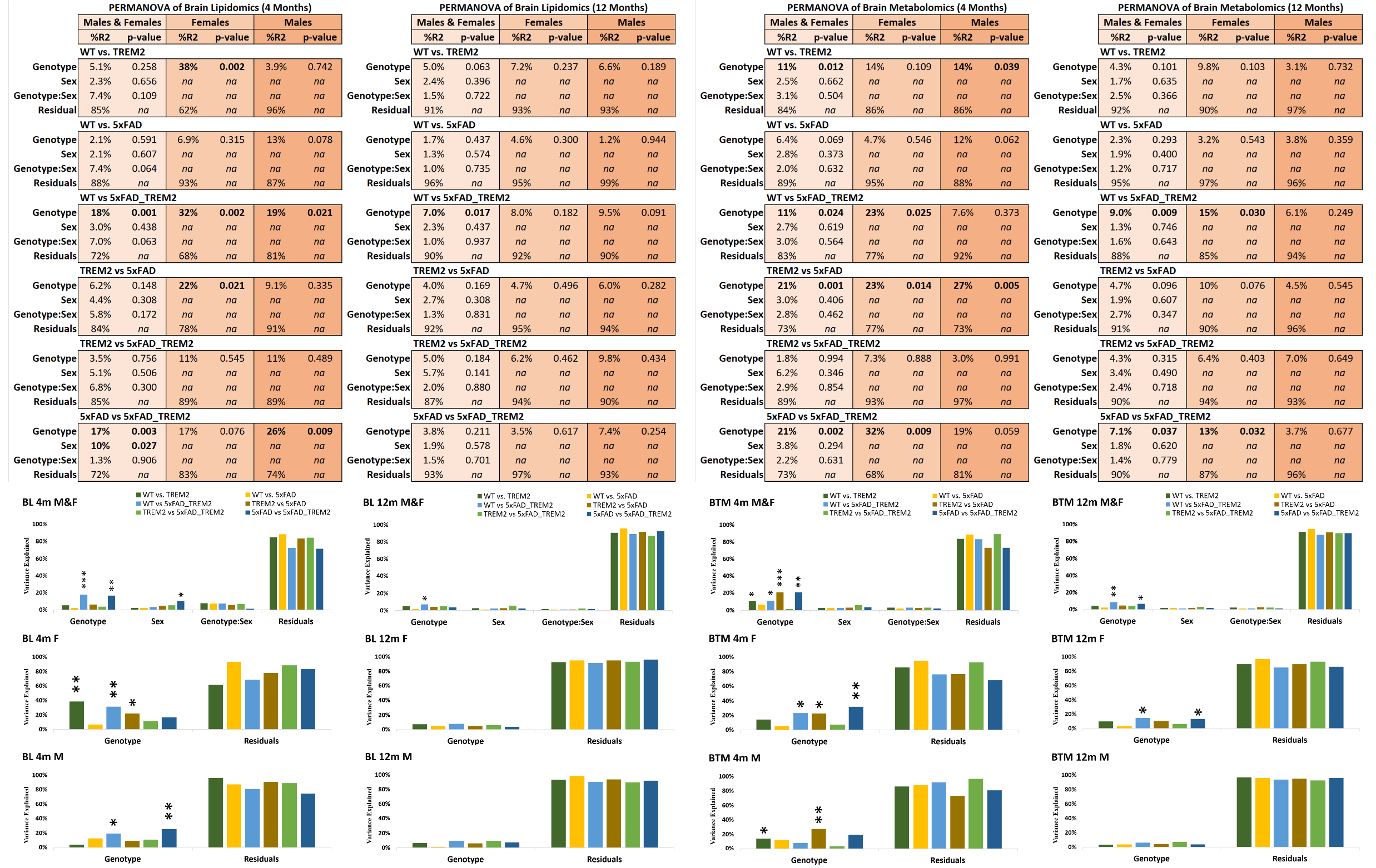
**

**Figure S5. PERMANOVA results for brain tissue.** Tables of PERMANOVA outputs (%R2 and p-value) for the brain lipidomic and polar metabolite datasets, stratified by Genotype (six pairwise comparisons between WT, *Trem2*^R47H^, 5xFAD, and 5xFAD, *Trem2*^R47H^ mice), Age (4-month and 12-month) and Sex (M&F, F, and M). The %R^2^ value represents the percentage of variance explained by each factor (Genotype and Sex). Significant effects are bolded. Below each table are the corresponding bar plots of %R^2^. Each bar represents a separate pairwise comparison between the four mouse genotype groups: WT vs. *Trem2*^R47H^ (dark green), WT vs. 5xFAD (yellow), WT vs. 5xFAD, *Trem2*^R47H^ (light blue), *Trem2*^R47H^ vs. 5xFAD (brown), *Trem2*^R47H^ vs. 5xFAD, *Trem2*^R47H^ (light green), and 5xFAD vs. 5xFAD, *Trem2*^R47H^ (dark blue). If the PERMANOVA p-value was significant for a specific comparison, an asterisk is shown above that bar. The number of asterisks represents the significance level (p = 0.001 (***), p <= 0.01 (**), and p <= 0.05 (*)). As with the plasma datasets, we generally observed that the variance and significance levels of each comparison decreased from 4-months to 12-months.

**
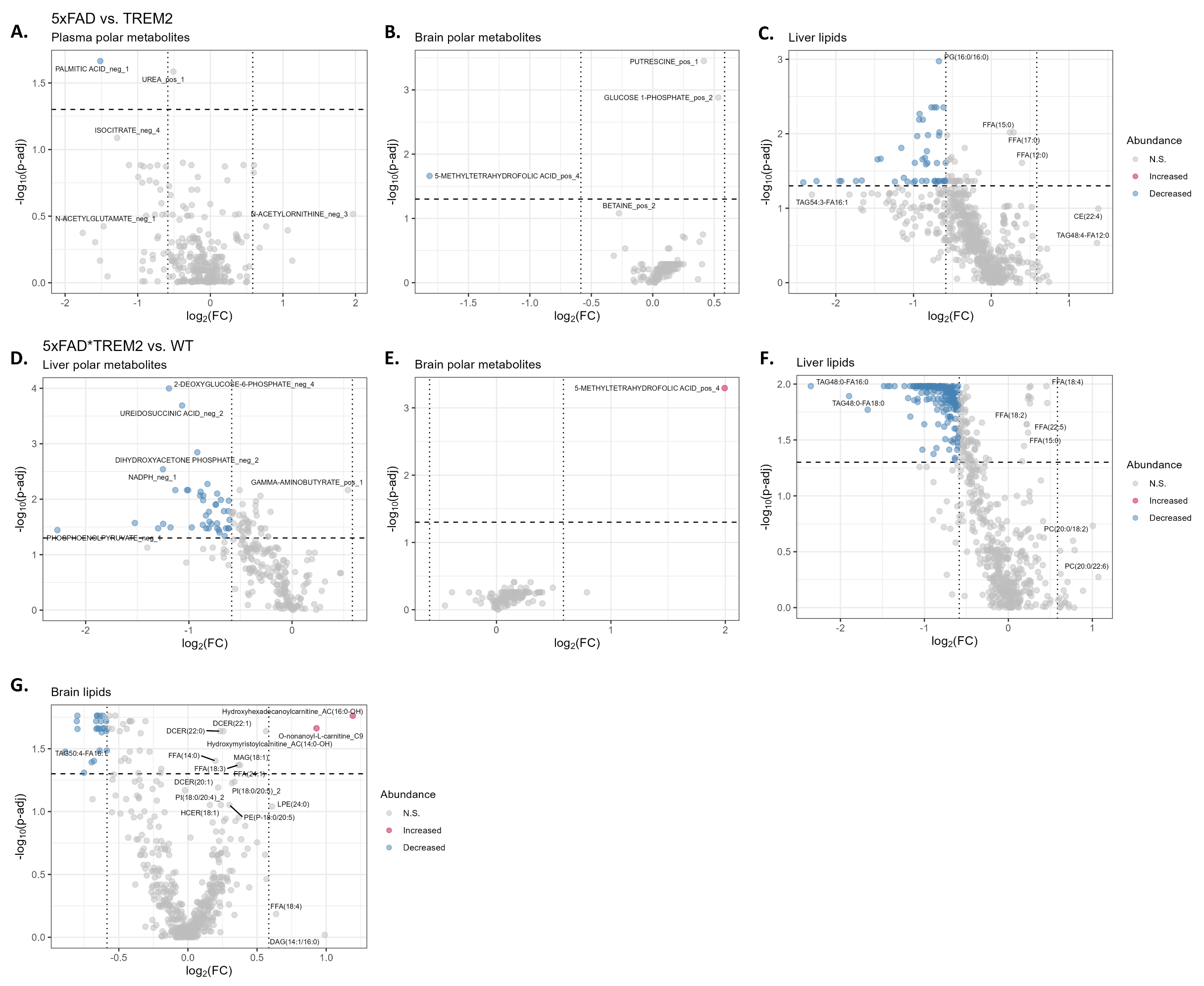
**

**Figure S6.** **Differential and synergistic effects of *Trem2^R47H^* variant and 5xFAD transgene.** Volcano plots in panels **(A, B, C)** show the significantly differentially abundant (DA) features between the *Trem2^R47H^* and 5xFAD groups across the combined 4- and 12-month ages. Significantly DA polar metabolites were identified in the plasma **(A)** and brain **(B)**, with no significant changes in the liver (not shown). Conversely, no lipids were significantly DA in the plasma or brain, with notable changes in the liver **(C)**. Volcano plots in panels **(D, E, F, G)** show the significantly DA features between the WT and 5xFAD, *Trem2*^R47H^ groups across the combined 4- and 12-month ages. Significant changes to both polar metabolites and lipids were observed in the liver **(D, F)** and brain **(E, G)**, but not in plasma.

**
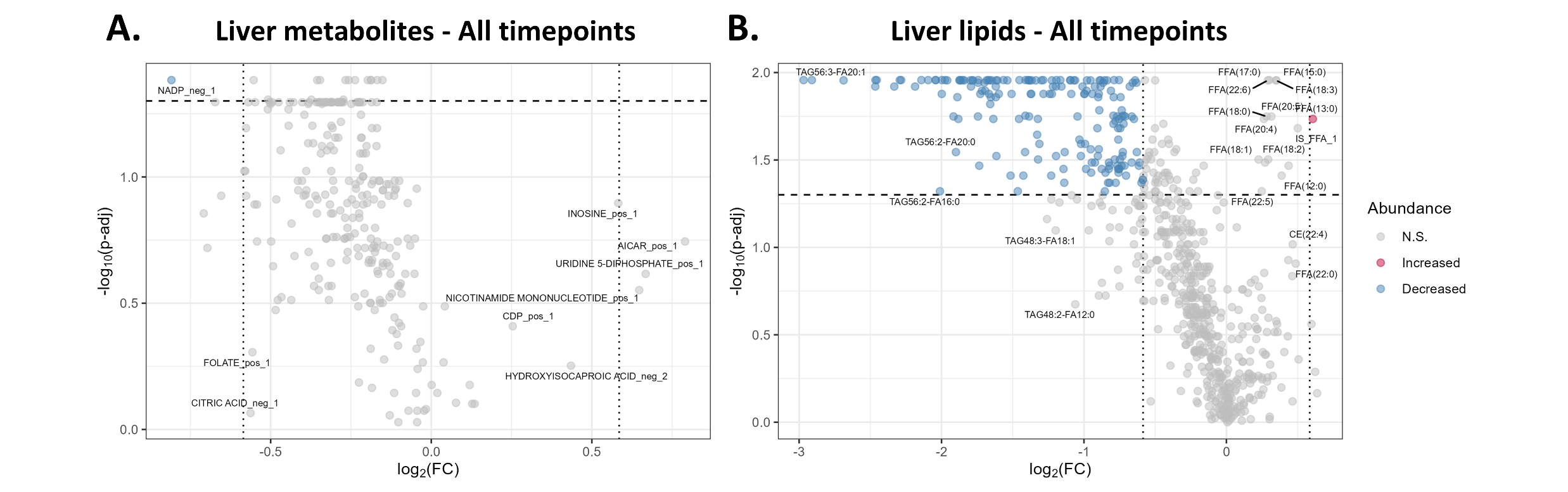
**

**Figure S7. Comparison of the *Trem2*^R47H^ and 5xFAD, *Trem2*^R47H^ groups across the 4- and 12-month timepoints.** Volcano plots of the DA metabolites (**A**) and lipids (**B**) for the *Trem2*^R47H^ and 5xFAD, *Trem2*^R47H^ comparison across the 4- and 12-month timepoints in the liver. Only a single metabolite (NADH) was DA, but an abundance of TAG’s were also DA, suggesting broad disruption of lipids in the liver due to the interaction of the *Trem2*^R47H^ variant on the 5xFAD background.

**
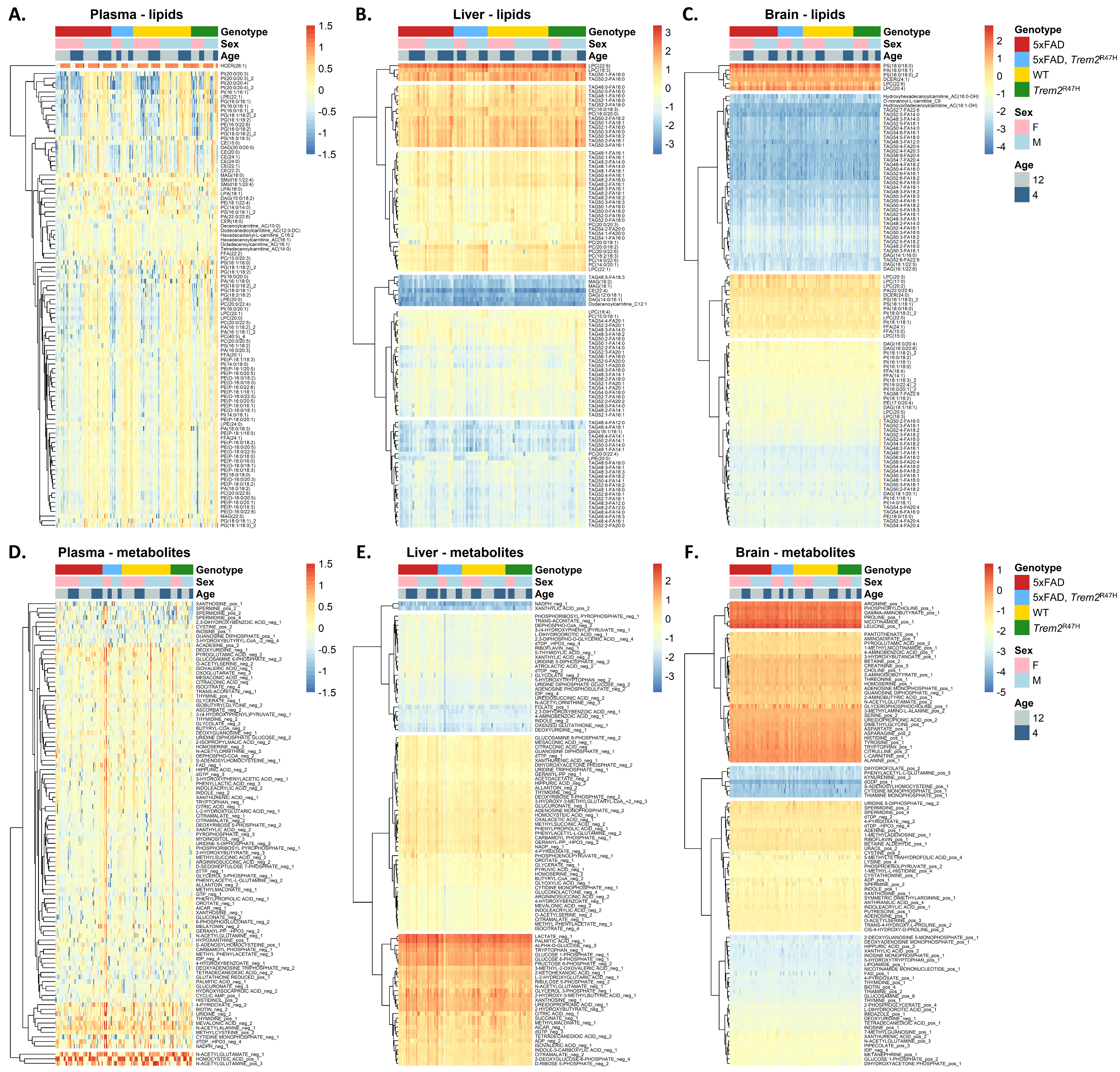
**

**Figure S8. Heatmaps of differences between 5xFAD and 5xFAD, *Trem2*^R47H^ groups for genotype, sex, and age.** Heatmaps illustrate the log10 abundances of metabolites detected in plasma **(A, D)**, liver **(B, E)**, and brain **(C, F)**. Abundances were measured for the lipidomic **(A, B, C)** and targeted (polar) metabolite datasets **(D, E, F)** separately. Metabolites and lipids were hierarchically clustered using a dendrogram based on Euclidean distances, while samples were organized in the order of sex, genotype, and age. Only the top 100 most differentially abundant metabolites and lipids between males and females, based on log2 fold change, were shown.

**
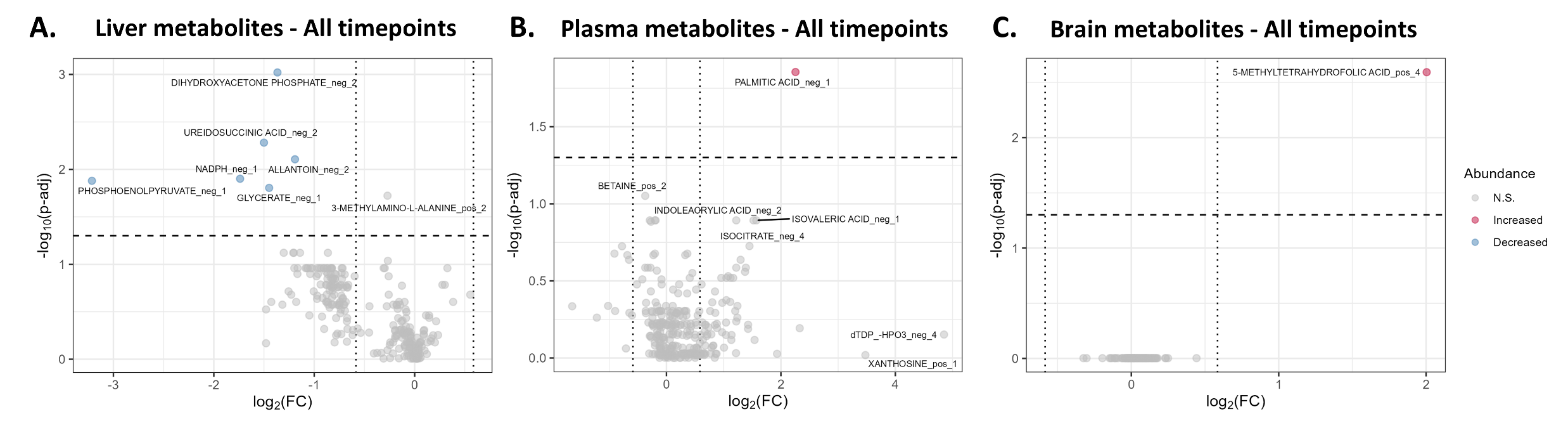
**

**Figure S9. Comparison of the 5xFAD and 5xFAD, *Trem2*^R47H^ groups across the 4- and 12-month timepoints.** Volcano plots show the significantly differentially abundant (DA) polar metabolites between the 5xFAD and 5xFAD, *Trem2*^R47H^ groups for the liver **(A),** plasma **(B)**, and brain **(C)** across the 4- and 12-month timepoints. There were 6 significantly DA polar metabolites in the liver, one in plasma, and one in the brain.


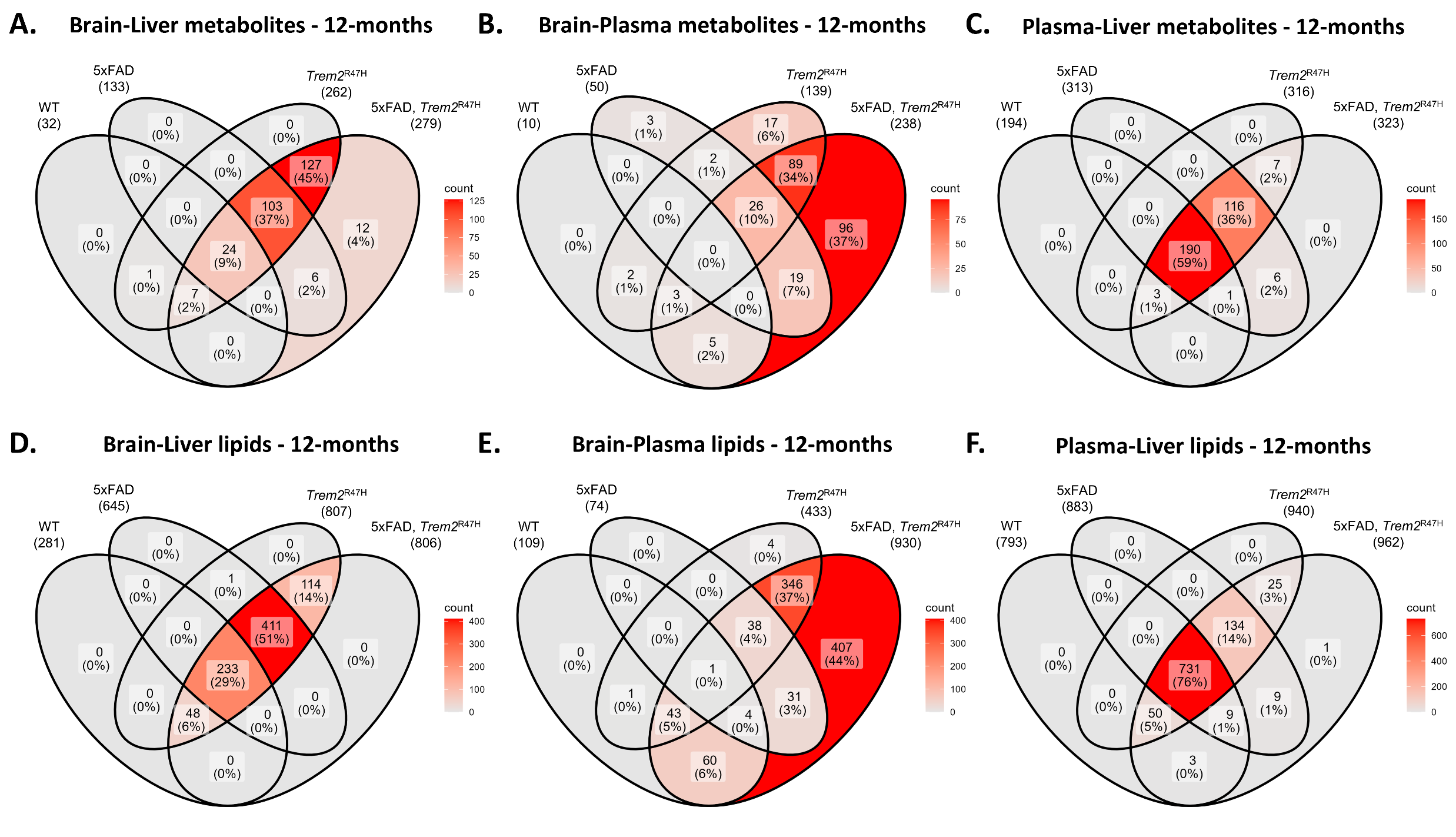


**Figure S10. Lipid and metabolite associations along the liver-plasma-brain axis at the 12-month timepoint.** Venn diagrams of strongly correlated (Spearman’s rho > 0.7) brain and liver metabolites **(A)**, brain and plasma metabolites **(B)**, plasma and liver metabolites **(C)**, brain and liver lipids **(D)**, brain and plasma lipids **(E)**, and plasma and liver lipids **(F)** across the four AD genotypes at the 12-month timepoint. The number of correlated analytes is indicated in parentheses below each genotype label. Color intensity increases with the number of analytes in each section.

**
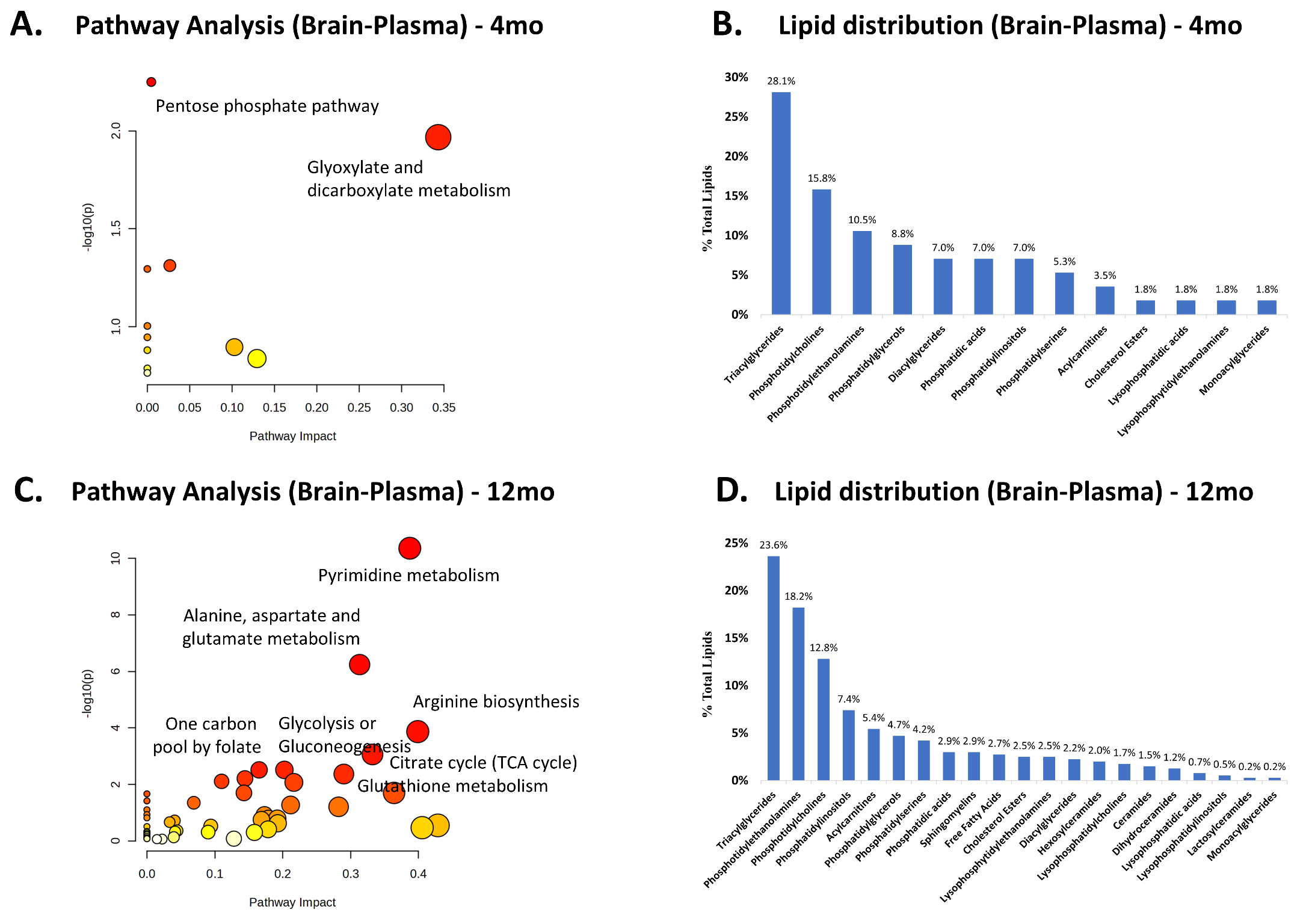
**

**Figure S11. Synergistic effects of *Trem2^R47H^* variant and 5xFAD transgene along the plasma-brain axis.** Pathway analysis maps in panels **(A, C)** show pathways enriched by the correlated polar metabolites at 4-months **(A)** and 12-months **(C)**. The color of the circles representing each pathway corresponds to their significance level, and their size corresponds to their Pathway Impact score, a metric that estimates the importance of each pathway relative to the global metabolic network. Bar graphs in panels **(B, D)** show the lipid distributions of correlated lipids at 4-months **(B)** and 12-months **(D)**.
